# Artificial intelligence in cancer imaging: Clinical challenges and applications

**DOI:** 10.3322/caac.21552

**Published:** 2019-02-05

**Authors:** Wenya Linda Bi, Ahmed Hosny, Matthew B. Schabath, Maryellen L. Giger, Nicolai J. Birkbak, Alireza Mehrtash, Tavis Allison, Omar Arnaout, Christopher Abbosh, Ian F. Dunn, Raymond H. Mak, Rulla M. Tamimi, Clare M. Tempany, Charles Swanton, Udo Hoffmann, Lawrence H. Schwartz, Robert J. Gillies, Raymond Y. Huang, Hugo J. W. L. Aerts

**Affiliations:** ^1^ Assistant Professor of Neurosurgery, Department of Neurosurgery, Brigham and Women’s Hospital, Dana‐Farber Cancer Institute Harvard Medical School Boston MA; ^2^ Research Scientist, Department of Radiation Oncology, Brigham and Women’s Hospital, Dana‐Farber Cancer Institute Harvard Medical School Boston MA; ^3^ Associate Member, Department of Cancer Epidemiology H. Lee Moffitt Cancer Center and Research Institute Tampa FL; ^4^ Professor of Radiology, Department of Radiology University of Chicago Chicago IL; ^5^ Research Associate, The Francis Crick Institute London United Kingdom; ^6^ Research Associate, University College London Cancer Institute London United Kingdom; ^7^ Research Assistant, Department of Radiology, Brigham and Women’s Hospital, Dana‐Farber Cancer Institute Harvard Medical School Boston MA; ^8^ Research Assistant, Department of Electrical and Computer Engineering University of British Columbia Vancouver BC Canada; ^9^ Research Assistant, Department of Radiology Columbia University College of Physicians and Surgeons New York NY; ^10^ Research Assistant, Department of Radiology New York Presbyterian Hospital New York NY; ^11^ Assistant Professor of Neurosurgery, Department of Neurosurgery, Brigham and Women’s Hospital, Dana‐Farber Cancer Institute Harvard Medical School Boston MA; ^12^ Research Fellow, The Francis Crick Institute London United Kingdom; ^13^ Research Fellow, University College London Cancer Institute London United Kingdom; ^14^ Associate Professor of Neurosurgery, Department of Neurosurgery, Brigham and Women’s Hospital, Dana‐Farber Cancer Institute Harvard Medical School Boston MA; ^15^ Associate Professor, Department of Radiation Oncology, Brigham and Women’s Hospital, Dana‐Farber Cancer Institute Harvard Medical School Boston MA; ^16^ Associate Professor, Department of Medicine Brigham and Women’s Hospital, Dana‐Farber Cancer Institute, Harvard Medical School Boston MA; ^17^ Professor of Radiology, Department of Radiology, Brigham and Women’s Hospital, Dana‐Farber Cancer Institute Harvard Medical School Boston MA; ^18^ Professor, The Francis Crick Institute London United Kingdom; ^19^ Professor, University College London Cancer Institute London United Kingdom; ^20^ Professor of Radiology, Department of Radiology Massachusetts General Hospital and Harvard Medical School Boston MA; ^21^ Professor of Radiology, Department of Radiology Columbia University College of Physicians and Surgeons New York NY; ^22^ Chair, Department of Radiology New York Presbyterian Hospital New York NY; ^23^ Professor of Radiology, Department of Cancer Physiology H. Lee Moffitt Cancer Center and Research Institute Tampa FL; ^24^ Assistant Professor, Department of Radiology, Brigham and Women’s Hospital, Dana‐Farber Cancer Institute Harvard Medical School Boston MA; ^25^ Associate Professor, Departments of Radiation Oncology and Radiology, Brigham and Women’s Hospital, Dana‐Farber Cancer Institute Harvard Medical School Boston MA; ^26^ Professor in AI in Medicine, Radiology and Nuclear Medicine, GROW Maastricht University Medical Centre (MUMC+) Maastricht The Netherlands

**Keywords:** artificial intelligence, cancer imaging, clinical challenges, deep learning, radiomics

## Abstract

Judgement, as one of the core tenets of medicine, relies upon the integration of multilayered data with nuanced decision making. Cancer offers a unique context for medical decisions given not only its variegated forms with evolution of disease but also the need to take into account the individual condition of patients, their ability to receive treatment, and their responses to treatment. Challenges remain in the accurate detection, characterization, and monitoring of cancers despite improved technologies. Radiographic assessment of disease most commonly relies upon visual evaluations, the interpretations of which may be augmented by advanced computational analyses. In particular, artificial intelligence (AI) promises to make great strides in the qualitative interpretation of cancer imaging by expert clinicians, including volumetric delineation of tumors over time, extrapolation of the tumor genotype and biological course from its radiographic phenotype, prediction of clinical outcome, and assessment of the impact of disease and treatment on adjacent organs. AI may automate processes in the initial interpretation of images and shift the clinical workflow of radiographic detection, management decisions on whether or not to administer an intervention, and subsequent observation to a yet to be envisioned paradigm. Here, the authors review the current state of AI as applied to medical imaging of cancer and describe advances in 4 tumor types (lung, brain, breast, and prostate) to illustrate how common clinical problems are being addressed. Although most studies evaluating AI applications in oncology to date have not been vigorously validated for reproducibility and generalizability, the results do highlight increasingly concerted efforts in pushing AI technology to clinical use and to impact future directions in cancer care.

## Introduction

Cancer, as a self‐sustaining and adaptive process that interacts dynamically with its microenvironment, continues to thwart patients, researchers, and clinicians despite significant progress in understanding its biological underpinnings. Given this complexity, dilemmas arise at every stage of cancer management, including reliable early detection; accurate distinction of preneoplastic and neoplastic lesions; determination of infiltrative tumor margins during surgical treatment; tracking of tumor evolution and potential acquired resistance to treatments over time; and prediction of tumor aggressiveness, metastasis pattern, and recurrence. Technological advances in medical imaging and minimally invasive biomarkers hold promise in addressing such challenges across the spectrum of cancer detection, treatment, and monitoring. However, the interpretation of the large volume of data that is generated by these advancements presents a barrage of new potential challenges.

As we learn more about the disease itself, we are learning more about the power of tools that are already available to us, which may be used in unprecedented ways. When a neoplastic lesion is initially detected, it needs to be distinguished from nonneoplastic mimickers and classified based on its predicted clinical course and biological aggressiveness to optimize the type and intensity of treatment. The widespread availability of computed tomography (CT) and magnetic resonance imaging (MRI) have fueled the incidental detection of lesions within the body with unclear clinical significance, which then initiates a cascade of observation, further testing, or empiric intervention. With treatment, which includes cytoreduction through surgery, elicitation of direct and indirect mechanisms of tumor kill through radiation, and pharmacotherapies, cancers may adapt to the stressors imposed, evolve, and recur. With the radiographic appearance of a lesion that increases in size after treatment, distinction has to be made between neoplasm or tissue response to injury. On recurrence, neoplastic lesions have been shown to harbor new molecular aberrations distinct from the primary tumor, which may confer resistance to medical or radiation therapies. This is compounded by the innate intratumoral heterogeneity of cancers at the time of initial diagnosis, which is increasingly demonstrated by research but difficult to capture in routine clinical pathological sampling and profiling. The demand for noninvasive imaging, as the most common method to track response to treatment and to suggest critical information about tumors themselves, has never been greater.

Traditional radiographic imaging evaluation of tumor relies upon largely qualitative features, such as tumor density, pattern of enhancement, intratumoral cellular and acellular composition (including the presence of blood, necrosis, and mineralization), regularity of tumor margins, anatomic relationship to the surrounding tissues, and impact on these structures. Size‐based and shape‐based measures of the tumor can be quantified in 1‐, 2‐, 3‐dimensional analyses. These qualitative phenotypic descriptions are collectively termed “semantic” features. In comparison, a rapidly evolving field called radiomics is enabling digital decoding of radiographic images into quantitative features, including descriptors of shape, size, and textural patterns.^1^ Recent advances in artificial intelligence (AI) methodologies have made great strides in automatically quantifying radiographic patterns in medical imaging data. Deep learning, a subset of AI, is an especially promising method that automatically learns feature representations from sample images and has been shown to match and even surpass human performance in task‐specific applications.^2^, ^3^ Despite requiring large data sets for training, deep learning has demonstrated relative robustness against noise in ground truth labels,^4^ among others. The automated capabilities of AI offer the potential to enhance the qualitative expertise of clinicians, including precise volumetric delineation of tumor size over time, parallel tracking of multiple lesions, translation of intratumoral phenotypic nuances to genotype implications, and outcome prediction through cross‐referencing individual tumors to databases of potentially limitless comparable cases. Furthermore, deep learning approaches promise greater generalizability across diseases and imaging modalities,^5^ robustness to noise,^6^ and reduction of errors—eventually leading to earlier interventions and significant improvements in diagnosis and clinical care. Although these studies remain largely in the preclinical research domain, the continued development of such automatic radiographic “radiomic” biomarkers may highlight clinically actionable changes in tumors and drive a paradigm shift in the discrimination of cancer over time.

At the dawn of this exciting technological transformation, we review the current evidence and future directions for AI approaches as applied to medical imaging in 4 common cancer types: lung, brain, breast, and prostate cancer. We describe clinical problems and limitations in cancer detection and treatment, how current methods are attempting to address these, and how AI can affect future directions.

## AI Applications in Cancer Imaging

The desire to improve the efficacy and efficiency of clinical care continues to drive multiple innovations into practice, including AI. With the ever increasing demand for health care services and the large volumes of data generated daily from parallel streams, the optimization and streamlining of clinical workflows have become increasingly critical. AI excels at recognizing complex patterns in images and thus offers the opportunity to transform image interpretation from a purely qualitative and subjective task to one that is quantifiable and effortlessly reproducible. In addition, AI may quantify information from images that is not detectable by humans and thereby complement clinical decision making. AI also can enable the aggregation of multiple data streams into powerful integrated diagnostic systems spanning radiographic images, genomics, pathology, electronic health records, and social networks.

**Figure 1:**
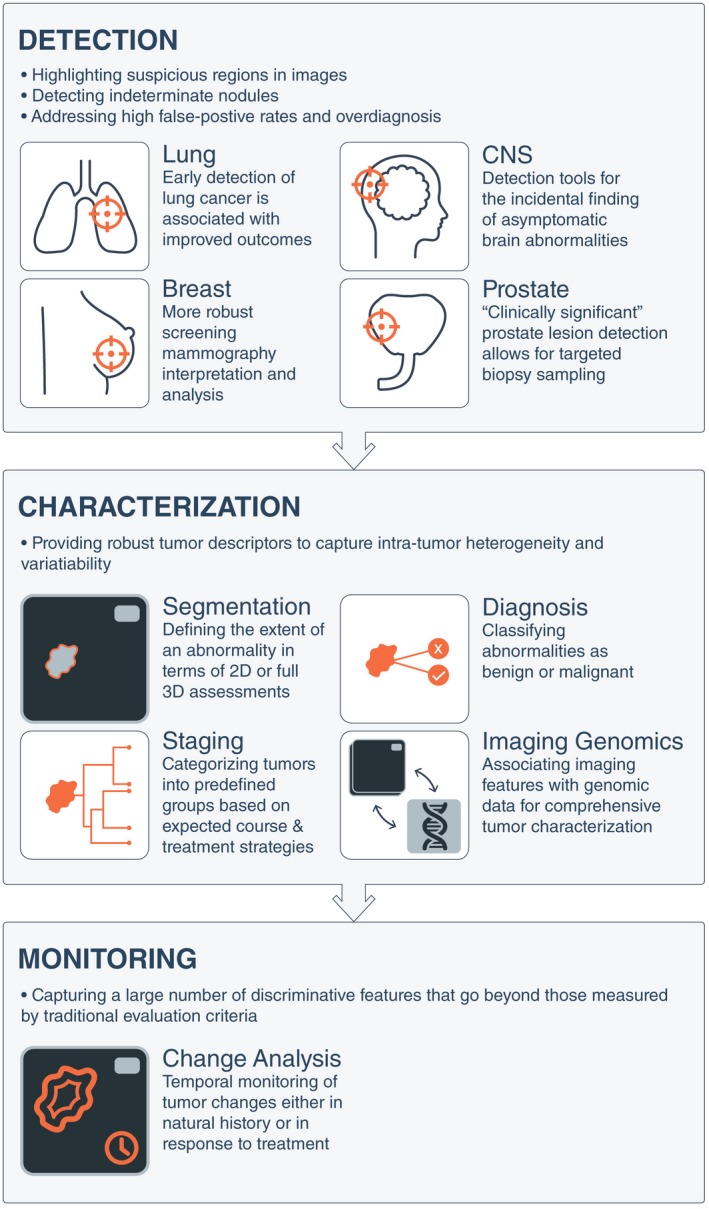
Artificial Intelligence Applications in Medical Imaging as Applied to Common Cancers. Artificial intelligence tools can be conceptualized to apply to 3 broad categories of image‐based clinical tasks in oncology: 1) detection of abnormalities; 2) characterization of a suspected lesion by defining its shape or volume, histopathologic diagnosis, stage of disease, or molecular profile; and 3) determination of prognosis or response to treatment over time during monitoring. 2D indicates 2‐dimensional; 3D, 3‐dimensional; CNS, central nervous system.

Within cancer imaging, AI finds great utility in performing 3 main clinical tasks: detection, characterization, and monitoring of tumors (Fig. 1). Detection refers to the localization of objects of interest in radiographs, collectively known as computer‐aided detection (CADe). AI‐based detection tools can be used to reduce observational oversights and serve as an initial screen against errors of omission.^7^ Formulated within a pattern‐recognition context, regions with suspicious imaging characteristics are highlighted and presented to the reader. CADe has been used as an auxiliary aide to identify missed cancers in low‐dose CT screening,^8^ detect brain metastases in MRIs to improve radiology interpretation time while maintaining high detection sensitivity,^9^ locate microcalcification clusters in screening mammography as an indicator of early breast carcinoma,^10^ and more generally has improved radiologist sensitivity for detecting abnormalities.^11^

Characterization broadly captures the segmentation, diagnosis, and staging of tumors. It also can extend to include prognostication based on a given ailment as well as outcome prediction based on specific treatment modalities. Segmentation defines the extent of an abnormality. This can range from basic 2‐dimensional (2D) measurements of the maximal in‐plane tumor diameter to more involved volumetric segmentations in which the entire tumor and possible surrounding tissues are assessed. Such information can be used in subsequent diagnostic tasks as well as dosage administration calculations during radiation planning. In current clinical practice, tumors are typically manually defined, with associated limitations including interrater bias,^12^ inconsistent reproducibility even among experts,^13^, ^14^ and consumption of time and labor. Although manually traced segmentation frequently is used as the basis for judging the accuracy of automated segmentation algorithms, it has the potential to neglect subclinical disease and restrict the region of analysis to human bias. AI has the potential to increase the efficiency, reproducibility, and quality of tumor measurements dramatically with automated segmentation. Finally, with the rapid expansion of computing speed and the increased efficiency of AI algorithms, it is likely that future analysis of cancer lesions will not require a separate segmentation step, and whole‐body imaging data could be evaluated directly by AI algorithm. A whole‐body approach also can allow an analysis of organ structures that may be pathological but are not apparent to human vision.

On radiologic data, the subsequent diagnosis of suspicious lesions as either benign or malignant ultimately results in a visual interpretation by radiologists. Clinically, human experience and expertise are applied to solving such problems using subjective, qualitative features. By comparison, computer‐aided diagnosis (CADx) systems use the systematic processing of quantitative tumor features, allowing for more reproducible descriptors. CADx systems have been used to diagnose lung nodules in thin‐section CT^15^ as well as prostate lesions in multiparametric MRI,^16^ in which inconsistencies in interpretation among human readers have been observed.^17^, ^18^ Characterization also includes staging, in which tumors are classified into predefined groups based on differences in their cancer’s appearance and spread that are informative for the expected clinical course and treatment strategies. The most widely used cancer staging system is the TNM classification,^19^, ^20^ with other schemes applied for specific organs such as the central nervous system (CNS). Recent studies have extended systems to perform staging by assessing tumor extent and multifocality in breast MRI,^21^ whereas others have developed automated lesion volume measurement tools in contrast‐enhanced magnetic resonance mammography (MRM).^22^

An additional level of characterization interrogates the biological characterization of tumors. The emerging field of “imaging genomics” correlates radiographic imaging features with biological data, including somatic mutations, gene expression, chromosome copy number, or other molecular signatures. The maturity of genomics analyses, from a data standpoint, provides synergistic opportunities for AI‐based imaging efforts.^23^

Finally, AI can play increasing roles in monitoring changes in a tumor over time, either in natural history or in response to treatment. Traditional temporal monitoring of tumors often has been limited to predefined metrics including tumor longest diameter measured through the established Response Evaluation Criteria in Solid Tumors (RECIST) and World Health Organization (WHO) criteria for estimating tumor burden and determining treatment response. In addition to being criticized as oversimplifying the complex tumor geometry captured through sophisticated imaging instruments,^24^ the generalizability and efficacy of such criteria have been questioned, as in the case of osseous lesions, for which chemotherapy—which has proven to improve survival—does not result in radiographic responses as measured by RECIST.^25^ AI‐based monitoring, however, is able to capture a large number of discriminative features across images over time that go beyond those measured by human readers. Although the seemingly disparate constituents of computer‐aided monitoring are active areas of research (computer‐aided registration of temporal images, segmentation, and diagnosis), the field is still in its infancy, with applications yet to surface.

In addition to imaging, other minimally invasive biomarkers also are being developed for cancer diagnosis and longitudinal tracking of disease. Most notably, liquid biopsies, or the analysis of circulating tumor DNA (ctDNA) released from tumor cells, provide a window into the current and dynamic state of a cancer^26^ and allows the tracking of disease progression or regression and monitoring for the emergence of targetable or resistance‐associated cancer mutations in near real‐time.^27^, ^28^, ^29^ Thus, it is conceivable that liquid biopsies, combined with radiomics profiling, may significantly improve cancer treatment through the noninvasive characterization of cancer biology for a more accurate assessment of prognosis and real‐time disease monitoring for the purposes of precision medicine.

**Figure 2:**
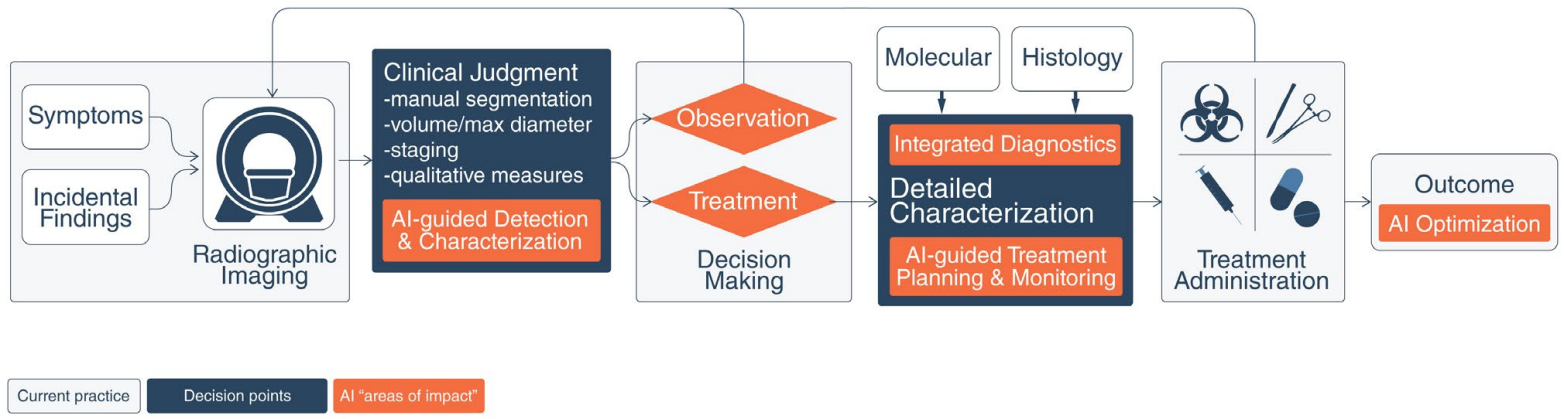
Potential Enhanced Clinical Workflow With Artificial Intelligence (AI) Interventions. The traditional paradigm for patients with tumors entails initial radiologic diagnosis of a mass lesion, a decision to treat or observe based on clinical factors and patient preference, a definitive histopathologic diagnosis only after obtaining tissue, molecular genotyping in centers with such resources, and determination of clinical outcome only after the passage of time. In contrast, AI‐based interventions offer the potential to augment this clinical workflow and decision making at different stages of oncological care. Continuous feedback and optimization from measured outcomes may further improve AI systems.

Within the clinic, the aforementioned AI interventions are expected to augment their respective current standard‐of‐care counterparts (Fig. 2). In addition to supporting clinicians with assistive information, multiple efforts also have demonstrated the utility of AI in the clinical decision‐making phases of the workflow.^30^ With AI‐based integrated diagnostics, combining molecular and pathological information with image‐based findings will add rich layers of intelligence to the findings, eventually leading to more informed decision making.

## Lung Cancer Imaging

Lung cancer is a leading cause of cancer‐related death among men and women globally.^31^ Despite improvements in survival over the last several decades for most cancer types, lung cancer is falling behind, mainly because the cancer is often well advanced, with limited treatment options by the time it is detected.^32^ The finding that the majority of patients who are diagnosed with lung cancer will die from their disease can be attributed to the late stage at diagnosis. Medical imaging and AI are expected to play an important role in improving the early detection and characterization of lung cancer by differentiating benign from malignant nodules. Because early stages are often curable, this could drastically improve patient outcomes, minimize overtreatment, and even save lives. Furthermore, AI also can enhance lung cancer staging and characterization for treatment selection, as well as monitoring treatment response (Table 1).^1^, ^33^, ^34^, ^35^, ^36^, ^37^, ^38^, ^39^, ^40^, ^41^

**Table 1:** Summary of Key Studies on the Role of Artificial Intelligence in Imaging of Lung Cancer, as Applied to Detection, Diagnosis, and Characterization, and Predicting Prognosis and Treatment Response

REFERENCE	TUMOR(S) STUDIED	APPLICATION	NO. OF CASES	IMAGING MODALITY	MACHINE LEARNING ALGORITHM	IMAGING FEATURE TYPE	TYPE OF VALIDATION	RESULTS
Cancer detection								
Hawkins 2016^33^	NSCLC	Risk of lung cancer in screening/early detection	600	CT	Random forests classifier	Predefined radiomic features	Independent validation within ACRIN 6684	AUC, 0.83
Liu 2017^34^	NSCLC	Predict lung cancer in indeterminate pulmonary nodules	172	CT	Multiple supervised technique	Semantic	Independent validation with single‐center data	AUC, 0.88; ACC, 81%; Sn, 76.2%; Sp, 91.7%
Ciompi 2017^35^	Benign vs malignant lung lesions	Predict lung cancers in screening	1411	CT	SVM	Deep learning radiomics	Independent validation with multicenter data	ACC, 73%
Diagnosis and characterization								
Yamamoto 2014^36^	NSCLC	Discriminate ALK+ from non‐ALK tumors	172	CT	Random forests classifier	Semantic	Independent validation with multicenter data	Discriminatory power for ALK+ status: Sn, 83.3%; Sp, 77.9%; ACC, 78.8%
Maldonado 2015^37^	Lung adenocarcinoma	Differentiate indolent vs aggressive adenocarcinoma	294	CT	Previously built CANARY model	Semantic (CANARY)	Independent validation with single‐center data	Progression‐free survival curve HR (*P* < .0001)
Grossmann 2017^38^	NSCLC	Predict molecular and cellular pathways	351	CT	SVM	Predefined radiomic features	Independent validation with multicenter data	Autodegration pathway prediction (AUC, 0.69; *P* < 10^−4^); prognostic biomarkers combining radiomic, genetic, and clinical information (CI, 0.73; *P* < 10^−9^)
Rios Velazquez 2017^39^	NSCLC	Predict mutational status	763	CT	Random forests classifier	Predefined radiomic features	Independent validation with multicenter data	EGFR+ and EGFR− cases (AUC, 0.69); EGFR+ and KRAS+ tumors (AUC, 0.80)
Predicting treatment response and prognosis								
Aerts 2014^1^	NSCLC, head and neck cancer	Prognostic biomarkers	1019	CT	Regression	Predefined radiomic features	Independent validation with multicenter data	Prognostic CI, 0.70; CI, 0.69
Coroller 2015^40^	Lung adenocarcinoma	Predict distant metastasis	182	CT	Regression	Predefined radiomic features	Independent validation with single‐center data	CI, 0.61; P = 1.79 × 10^−17^
Sun 2018^41^	NSCLC	Predict the immune phenotype of tumors and clinical outcomes	491	CT	Regression	Predefined radiomic features	Independent validation with multicenter data	AUC, 0.67; 95% CI, 0.57‐0.77; *P* = .0019

Abbreviations: ACC, accuracy; ACRIN, American College of Radiology Imaging Network; ALK+, anaplastic lymphoma kinase positive; AUC, area under curve; CANARY, Computer‐Aided Nodule Assessment and Risk Yield; CI, concordance index; CT, computed tomography; EGFR+/EGFR−, epidermal growth factor receptor positive/negative; HR, hazard ratio; KRAS, KRAS proto‐oncogene, guanosine‐triphosphatase; NSCLC, non‐small cell lung cancer; Sn, sensitivity; Sp, specificity; SVM, support vector machine.

### Clinical Applications of AI in Lung Cancer Screening

Until recently, a method to detect early‐stage lung cancer has been elusive even among high‐risk populations. The National Lung Screening Trial (NLST) demonstrated that screening with low‐dose CT (LDCT) was associated with a significant 20% reduction in overall mortality among high‐risk current and former smokers.^32^ Lung cancers identified at an early stage, whether by LDCT screening or incidentally, are more amenable to surgical cure and improved survival outcomes compared with lung cancers that are detected upon presentation with clinical symptoms, which are more frequently at a later stage of disease.^42^ Although the emergence of immune checkpoint inhibitors and targeted therapies have demonstrated durable long‐term survival in subsets of patients, not all patients benefit from such treatment modalities; thus, early detection has the benefit of improving patient survival and may limit the need for extensive treatment. On the basis of these NSLT findings, annual LDCT is now recommended for high‐risk individuals and is second only to primary prevention (smoking cessation) for mitigating lung cancer mortality, especially for those who have quit smoking but remain at risk. Although the NLST demonstrated a clear benefit for reducing all‐cause mortality, many limitations are associated with the early detection of lung cancer that could be enhanced with advanced computational analyses.^32^, ^43^, ^44^, ^45^, ^46^ In the sections below, we describe current problems and limitations in lung cancer screening, how conventional methods are attempting to overcome these limitations, and how AI can improve these areas.

**Figure 3:**
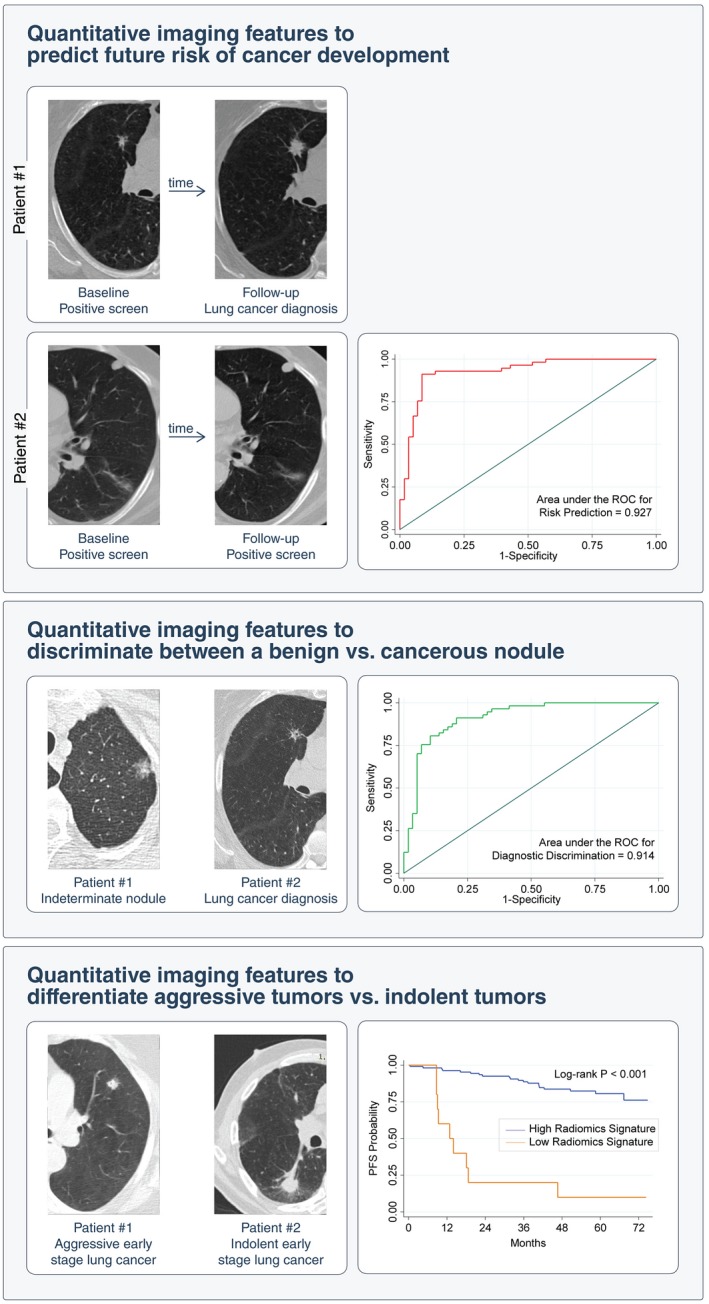
Clinical Applications of Artificial Intelligence in Lung Cancer Screening on Detection of Incidental Pulmonary Nodules. Imaging analysis shows promise in predicting the risk of developing lung cancer on initial detection of an incidental lung nodule and in distinguishing indolent from aggressive lung neoplasms. PFS indicates progression‐free survival; ROC, receiver operating characteristic.

Lung cancer screening frequently identifies large numbers of indeterminate pulmonary nodules, of which only a fraction are diagnosed as cancer (Fig. 3). In the NLST, 96.4% of the pulmonary nodules identified in LDCT screens were not cancerous. Currently, there are no established approaches to classify whether these nodules are cancerous or benign. Another potential harm of lung cancer screening is the overdiagnosis of slow‐growing, indolent cancers, which may pose no threat if left untreated. As such, it is imperative that overdiagnosis needs to be recognized, identified, and significantly reduced.^32^ Next, if a nodule is detected, clinical guidelines provide for the evaluation and follow‐up of nodules^47^ but do not offer decision tools for diagnostic discrimination and to predict risk and the probability of future cancer development. Although conventional biostatistics and machine‐learning approaches have been used to address many of the limitations in lung cancer screening, AI has the potential to supplant such approaches to identify biomarkers that reduce imaging false‐positive results and more accurately differentiate between benign and cancerous nodules. This can lead to a more quantitative prediction of lung cancer risk and incidence, leading to robust, better defined clinical decision guidelines.

The majority of indeterminate pulmonary nodules are incidentally detected (ie, they are not encountered during screening but in routine cross‐sectional imaging for other diagnostic indications, such as CT angiography),^48^ and pose a dilemma to patients and their providers. Annually, more than 1.5 million Americans are diagnosed with an incidentally detected nodule^49^; whereas most of these nodules are benign granulomas, up to 12% may be malignant.^50^ The Fleischner Society^51^ and the American College of Radiology Lung CT Screening Reporting and Data System (Lung‐RADS)^52^ provide recommendations for the follow‐up and management of these incidentally detected nodules, which usually require follow‐up imaging between 3 and 13 months to confirm growth before intervening with more invasive diagnostics (eg, biopsy). These systems are “semantic,” in that they describe features that are commonly used in the radiology lexicon to describe regions of interest by human experts. Because they are scored manually, there is high potential for large inter‐reader variability.^53^ In a recent study, a model incorporating 4 quantitatively scored semantic features (short‐axis diameter, contour, concavity, and texture) conferred an accuracy of 74.3% to distinguish malignant from benign nodules in the lung cancer screening setting.^34^ A separate study was conducted to identify semantic features from small pulmonary nodules (less than 6 mm) to predict lung cancer incidence in the lung cancer screening setting and the revealed final model yielded an area under the curve of the receiver operating characteristic of 0.930 based on total emphysema score, attachment to vessel, nodule location, border definition, and concavity.^54^ Although there was an imbalance between malignant and benign nodules in the aforementioned analyses, these studies provide compelling evidence for the utility of semantic features in lung cancer screening. As with nodules detected in the lung cancer screening setting, the standard of care for incidental pulmonary nodules lacks accurate decision tools for predicting malignancy versus benign disease and indolent versus aggressive behavior. Thus, the appropriate management of incidental pulmonary nodules is dictated by the probability of cancer and the potential aggressiveness of its behavior. Prediction of the nature of a nodule may justify diametrically opposing strategies, such as biopsy versus observation. Erroneous prediction carries significant consequences, including a risk of premature mortality from delayed intervention on the one hand and morbidity and mortality resulting from invasive testing on the other. Lung cancer screening also detects cancers that exhibit a wide spectrum of behaviors: some are clinically indolent, and others are aggressive, mandating prompt treatment. One study estimated that greater than 18% of all lung cancers detected by LDCT in the NLST seem to be indolent.^43^

In 2017, the Arnold Foundation supported a $1 million prize for the automated lung cancer detection and diagnosis challenge. In this challenge, thousands of annotated CT images from The Cancer Imaging Archive at the National Cancer Institute (NCI) were provided to the community to train and validate their models. All of the top teams used convolutional neural networks (CNNs) to automatically both detect and diagnose lesions, and the winners had to make their network model publicly available.^55^ The winning team reported a high performance (log loss = 0.399; in which a perfect model would have a log loss of 0). Although this is encouraging, it is notable that the winning networks require more detailed evaluation of their performance in clinical settings. Furthermore, there was a significant bias, with a 50% cancer prevalence in this challenge, which was higher than the 4% prevalence in a screening population with indeterminate nodules. Although this challenge identified promising methods, it is likely that significant fine tuning will be required before they can have any clinical use.

Although the incidence of lung cancer is declining in the United States and most Western nations,^56^ lung cancer will remain a major public health burden for decades to come. Even after smoking cessation, former smokers remain at increased risk, especially compared with lifetime never smokers, of developing lung cancer. Therefore, improvements in lung cancer screening will remain relevant and important to improve patient outcomes of this disease. As lung cancer imaging research has evolved from conventional biostatics, to machine learning, to deep learning, we contend that AI could emerge next to develop clinically adoptable approaches, precisely identify those at risk, improve risk prediction of future cancer incidence, discriminate malignant from nonmalignant nodules, and distinguish indolent tumors versus biologically aggressive cancers.

### Characterizing Lung Cancers Using Imaging

Lung cancers exhibit a wide spectrum of behaviors, with some that are clinically indolent and others that are aggressive, mandating prompt treatment. Although there are prognostic factors associated with better survival (such as female sex, tumors harboring an epidermal growth factor receptor [*EGFR*] mutation, early‐stage disease, no regional lymph node involvement, and a good performance status)^57^ as well as factors associated with poor survival (eg, poor pulmonary function, the presence of cardiovascular disease, male sex, current smoking status, advanced age, and late‐stage tumor),^58^, ^59^, ^60^, ^61^, ^62^ these factors have limited clinical utility to address the heterogeneous, dynamic nature of cancer as a “moving target.“ Specifically, a tumor lesion is constantly evolving and diversifying, modifying its phenotype and genomic composition and, through metastatic spread, even its location. This is even truer when subjected to the selection pressure of therapeutic intervention, in which cancer evolution rapidly explores and exploits resistance mechanisms, potentially even aided by the mutagenic nature of systemic cytotoxic chemotherapy, leaving the treating oncologist chasing a constantly changing disease.^63^, ^64^, ^65^

Image‐based biomarkers, conversely, can noninvasively and longitudinally capture the radiographic phenotype and characterize the underlying pathophysiology of a tumor. Because of the ease of clinical implementation, size‐based measures, such as the longest diameter of a tumor (eg, RECIST and WHO), are widely used for staging and response assessment. However, sized‐based features and stage of disease have limitations, as these metrics are associated with marked variability in outcomes and response. As such, research efforts to identify semantic features and automatic radiomic features to predict the outcomes of patients with lung cancer have been successful.^1^, ^40^, ^66^, ^67^, ^68^ For instance, the CANARY tool (Computer Aided Nodule Assessment and Risk Yield)^37^ offers semantic‐based risk stratification to identify a potentially vulnerable subset of lung adenocarcinomas that harbor a more aggressive course. Preliminary work has indicated that AI can quantify radiographic characteristics about the tumor phenotype automatically and that this information is significantly prognostic in several cancer types, including lung cancer (*P* < 3.53 × 10^−6^)^69^; in addition, it is associated with distant metastasis in lung adenocarcinoma (*P* = 1.79 × 10^−17^),^40^ tumor histologic subtypes (*P* = 2.3 × 10^‐7^),^68^ and underlying biological patterns, including somatic mutations^39^ and gene expression profiles.^38^

### Assessing Intratumor Heterogeneity Through Medical Imaging

Medical imaging also can play an important role in quantifying the intratumor characteristics of lung cancer. Sequencing studies in which multiple, independent samples from the same tumor have been analyzed have demonstrated that intratumor heterogeneity (ITH) is a common feature in solid tumor cancers.^70^ A tumor consists of billions of independent cancer cells. Low levels of DNA damage or changes in epigenetic regulation are introduced at each cell division, causing slight changes to the cancer cell genome that increase over time. When a change induces a selective advantage in a particular microenvironment, clonal expansion can give rise to a cancer subclone, with all the cancer cells sharing a single, recent, common ancestor. Genomic ITH, defined as the coexistence of independent cancer subclones within the same tumor, is associated with a poor prognosis in non–small cell lung cancer (NSCLC) and clear cell renal cancer.^63^, ^70^, ^71^, ^72^, ^73^ However, tumor subclones may be spatially separated and can carry significantly different mutation loads, ranging from highly homogeneous to greater than 8000 heterogeneous mutations differing between individual regions in the same tumor.^63^, ^65^

**Figure 4:**
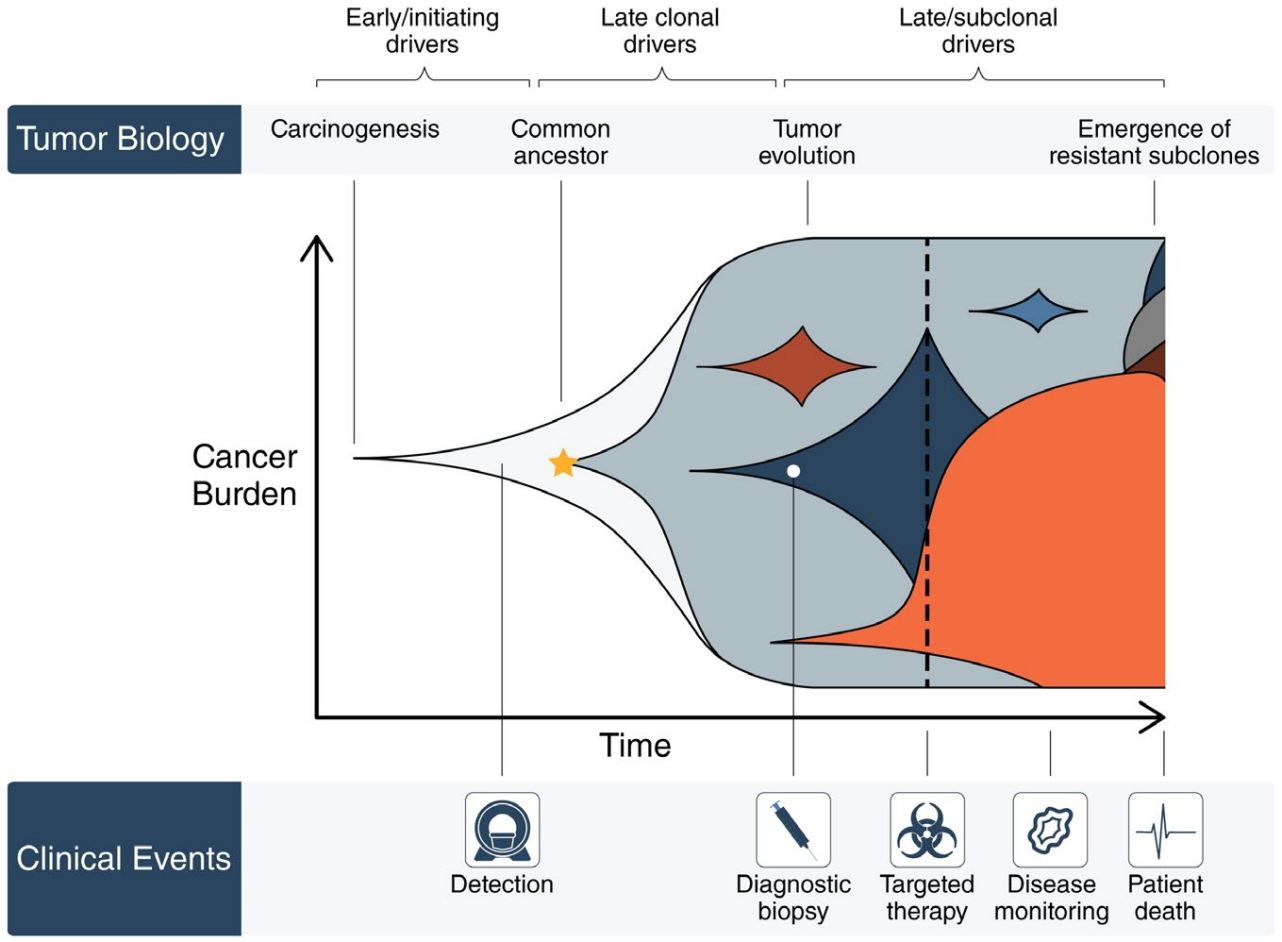
Applications of Noninvasive Monitoring During the Course of Cancer Evolution. Cancers share a common theme in developing intratumoral heterogeneity during their natural history. The presence of subclones (represented by different colors) confers significant implications in the response to treatment and may be difficult to capture through standard biopsies. Imaging and blood biomarkers during disease monitoring offer a potential technological solution for detecting the presence of intratumoral heterogeneity through space and time and thereby, perhaps, a direct change in therapeutic strategies.

ITH analysis has indicated that, although targetable somatic alterations may appear to be clonal in a single tumor biopsy, they may be entirely absent in additional biopsies from different regions of the same tumor.^62^, ^73^, ^74^ This evidence that phenotypic diversification exists within tumors has ramifications for the application of precision medicine techniques based on the molecular characterization of tissue from single‐region biopsies. Because the targets identified in single‐tumor biopsies may be subclonal, therapies against them would be effective only against a subset of the cancer cells, leaving cancer subclones without the target unharmed (Fig. 4). Different strategies have been proposed to quantify ITH in the clinical setting, including multiregion sequencing of the primary tumor, analysis of circulating tumor DNA, and use of medical imaging data.^26^, ^70^, ^75^ Unfortunately, although multiregion sequencing provides improved measures of the extent of ITH compared with single‐sample analysis,^76^, ^77^ it requires a high‐quality tumor specimen of sufficient size and remains subject to potential sampling bias, with the potential to miss important cancer subclones because of incomplete sampling of the tumor in its entirety.

Medical imaging can play an important role in quantifying the intratumor characteristics of lung cancer and improve the ability to capture and quantify ITH. Furthermore, because evolutionary fitness is contextual and depends on the particular microenvironment, it is likely that these environments can be identified by imaging.^78^ Similar to most tumor‐based biomarkers, there are many limitations, in that they can be subjective to sampling bias because of the heterogeneous nature of tumors and the requirement of tumor specimens for biomarker testing, and the assays often can be timely, expensive, and require large amounts of tissue or tissue analytes.^79^ In contrast, image‐based features, guided by AI, are available in real time from standard‐of‐care images, do not require timely (and often expensive) laboratory assay testing, are not subject to sampling bias and artifact, and, of course, are noninvasive. And image‐based features represent the phenotype of the entire tumor in 3 dimensions (3D), and not just the portion that was subjected to biomarker testing (ie, from a biopsy).^79^

### AI for Assessing Response to Targeted Therapies and Immunotherapies

The success of quantitative imaging endpoints based on the RECIST criteria paved the way for the development of AI in oncology, because the widespread adoption of these endpoints as early indicators of survival in clinical trials generated large data sets of CT images with clinical metadata. Retrospective analysis of these clinical trial data sets has been invaluable in meeting the need of AI for big data to enable training and validating AI algorithms, which otherwise might have been prohibited by the expense and effort necessary to generate these data sets from scratch. In part because of the success of RECIST, quantitative CT analysis is now the workhorse of contemporary oncology,^80^ creating immediate translational potential for AI predictive models.

The strengths of AI are well suited to overcome the challenge posed by the current generation of targeted and immunotherapies, which can produce a clear clinical benefit that is poorly captured by endpoints based on RECIST. These endpoints rely on the assumption that a successful response to therapy will be reflected by tumor shrinkage and, in particular, the measurement of response based on tumor diameter assumes that tumors are spherical and undergo uniform spatial change after treatment. Targeted therapies and immunotherapies lead to novel patterns of response that confound current RECIST‐based endpoints and may contribute to the high failure rate of clinical trials and the cost of drug development. Thus, the ability of AI to quantify the biological processes associated with response other than size answers an urgent need in the field.

Currently, response prediction for targeted and immunotherapies is based on biomarkers for immunogenic tumor microenvironment (eg, programmed cell death ligand 1 [PD‐L1] expression) and mutational status (eg, *EGFR*). These are acquired via biopsy, which is invasive, difficult to perform longitudinally, and limited to a single region of a tumor. The predictive value of PD‐L1 expression also may be limited. For example, in the KEYNOTE‐189 clinical trial, immunotherapy with pembrolizumab in combination with standard chemotherapy produced a survival benefit in all patients regardless of PD‐L1 expression, even among those with a PD‐L1 tumor proportion score less than 1%, which should indicate a small chance of benefit.^81^

A growing body of evidence suggests that AI could assess response to immunotherapy through recognition of radiomic biomarkers associated with response. Imaging phenotype was associated with overall survival (OS) in patients with NSCLC after second‐line treatment with anti‐PD1 (nivolumab). In this study, OS was predicted significantly (*P* = .005) by 2 radiomics features at baseline: region dissimilarity (hazard ratio [HR], 0.11; 95% confidence interval [95% CI], 0.03‐0.46 [*P* = .002]) and entropy (HR, 0.20; 95% CI, 0.06‐0.67 [*P* = .009]), which indicate a more heterogeneous primary lung tumor with irregular patterns of intensities on contrast‐enhanced CT. Another lung cancer study demonstrated that the prognosis of OS was improved by adding genomic and radiomic information to a clinical model, leading to an increase from a 95% CI of 0.65 (Noether *P* = .001) to a 95% CI of 0.73 (*P* = 2 × 10^−9^), and that the inclusion of radiomic data resulted in a significant increase in performance (*P* = .01).^38^ These findings indicate that radiomic and genomic biomarkers are complementary, creating a potential role for AI to elucidate predictive associations between their combined data. Although machine learning has been deployed to genetically classify lung cancer based on the identification of patterns in microarray gene expression,^82^ its use to detect radiomic‐genomic correlations predictive of outcome remains understudied.^38^

AI analysis of quantitative imaging data also may improve the assessment of response to targeted therapy. A decrease in fluorodeoxyglucose uptake by NSCLC tumors treated with bevacizumab, a monoclonal antibody against vascular endothelial growth factor (VEGF), identified more patients who responded to treatment than conventional CT criteria (73% vs 18%); in that study, neither positron emission tomography (PET) nor CT was associated with OS (PET, *P* = .833; CT, *P* = .557).^83^ Currently, predicting response to targeted therapy is driven largely by biopsy to assay the status of the mutation being targeted. AI predictive models could supplement this by identifying imaging phenotypes that are associated with mutational status. This approach has the advantage of being able to characterize the mutational status of all tumors repeatedly and noninvasively, not merely at the site of the biopsy, which can avoid the lack of predictive power associated with intratumoral heterogeneity and the emergence of distinct acquired resistance mechanisms in separate metastases within the same patient. Support for this approach comes from a quantitative imaging study of patients with NSCLC who were treated with gefitinib. Those results indicated that *EGFR* mutation status could be significantly predicted by the radiomic feature Laws‐Energy (area under the curve [AUC] = 0.67; *P* = .03).^84^

Biomarkers must be objectively and reproducibly measurable to serve as criteria for response assessment. AI affords high objectivity through its ability to characterize complex patterns within tumor images without the interobserver variability associated with visual assessment by human experts. Understanding the measurement error of radiomic features is important to establish the reproducibility of AI predictive models based on them. Different tumor segmentation algorithms introduce variance known to affect the calculation of radiomic features and thus perhaps the performance of AI techniques, which require semiautomatic segmentation.^85^ Imaging settings, including CT scanners, slice thickness, and reconstruction kernels, also affect the calculation of radiomic features.^86^, ^87^ Variation in these settings exists within clinical practice and clinical trials and may affect the power and reproducibility of biomarkers developed by AI. The training and validation of CNNs may reduce this effect by selecting predictive features that are reproducible and discarding those that vary between image sets, but this needs to be proven. There is also a tension between the rapid pace of development in the AI field and the need for clinical trial endpoints to maintain historical consistency and achieve validation in large data warehouses before criteria are updated (eg, from RECIST 1.0 to 1.1). Continued progress in the size and appropriateness of public domain cancer data sets is necessary to meet the latter requirement.

## CNS Tumor Imaging

CNS tumors span a broad spectrum of pathologies, and perhaps are more diverse than tumors of any other organ system in the body. Among tumors arising from or seeding in brain parenchyma, metastases from systemic cancers and gliomas predominate. In addition, a multiplicity of tumors arising from non‐neural tissues that abut the brain are commonly encountered and must be considered within CNS tumors, including meningiomas, pituitary tumors, schwannomas, and lesions of the skull. This variegated diorama of diagnoses poses unique demands on clinicians for the accurate assessment of imaging.

Three main challenges currently exist during the evaluation of radiologic studies for CNS tumors: 1) accurate diagnosis of the type and extent of disease is tantamount to clinical decision making; 2) reliable tracking of neoplastic disease over time, especially after treatment with its associated effects on surrounding neural tissue, which may acquire signal characteristics difficult to distinguish from tumor; and 3) the ability to extract genotype signatures from the phenotypic manifestation of tumors on imaging, as the impact of molecular taxonomy becomes increasingly appreciated in influencing tumor behavior and clinical outcome.

The traditional paradigm for patients with tumors entails initial radiologic diagnosis of a mass lesion, a decision to treat or observe based on clinical factors and surgeon or patient preference, a definitive histopathologic diagnosis only after obtaining tissue, molecular genotyping in centers with such resources, and the determination of clinical outcome only after the passage of time (Fig. 2). Accurate extrapolation of pathologic and genomic data from imaging data alone, similar to what is being developed in the field of imaging genomics, would disrupt this classic paradigm to improve the guidance of patients using more informed data upfront. Imaging genomics also may shed light on reasons for treatment success and failure across a patient population and in multi‐institutional clinical trials across heterogeneous populations. Furthermore, in locations around the globe with scarce access to expert neuroradiologists, limited encounters with rare CNS tumors, or a lack of molecular profiling, computational analysis of imaging through shared network algorithms offers a potentially valuable resource to improve care to all patients with brain tumors.

### Diagnostic Dilemmas in CNS

Imaging plays an important role in the initial diagnosis of brain tumors and is a routine part of both initial and subsequent evaluation. The complex imaging features of brain tumors, as well as the frequent genetic heterogeneity within tumor types and the invasive nature of the procedures needed to obtain a tissue diagnosis, give rise to diagnostic dilemmas in this field (Table 2).^88^, ^89^, ^90^, ^91^, ^92^, ^93^, ^94^, ^95^, ^96^, ^97^, ^98^, ^99^, ^100^, ^101^, ^102^, ^103^

**Table 2:** Summary of Key Studies on the Role of Artificial Intelligence in the Imaging of CNS Tumors, as Applied to Diagnosis, Biologic Characterization, Monitoring Treatment Response, and Predicting Outcome

REFERENCE		TUMOR(S) STUDIED	APPLICATION	NO. OF PATIENTS	IMAGING MODALITY	MACHINE LEARNING ALGORITHM	IMAGING/RADIOMIC FEATURE TYPE	TYPE OF VALIDATION^a^	PERFORMANCE
Diagnosis									
Fetit 2015^88^		Medulloblastoma, pilocytic astrocytoma, ependymoma	Classification of CNS tumor subtype	48	MRI conventional	Multiple supervised techniques	Texture	Leave‐one‐out cross‐validation, single center	AUC, 0.91‐0.99
Coroller 2017^89^		Meningioma	Differentiate grade 1 vs grade 2‐3	175	MRI conventional	Random forest	Radiomic and semantic features	Independent validation with single‐center data	AUC, 0.76‐0.86
Zhang 2017^90^		Glioma (WHO grade 2‐4)	LGG (WHO grade 2) vs HGG (grade 3‐4)	120	MRI conventional, perfusion, diffusion, permeability maps	SVM	Histogram, texture	Leave‐one‐out cross validation	ACC, 0.945
Zhang 2018^91^		Pituitary adenoma	Null cell adenoma vs other subtypes	112	MRI conventional	SVM	Intensity, shape, size, texture	Independent validation with single‐center data	AUC, 0.804
Kang 2018^92^		Glioblastoma, lymphoma	Classify glioblastoma vs lymphoma	198	MRI conventional, perfusion, diffusion maps	Multiple supervised techniques	Volume, shape, texture	Independent validation with multicenter data	AUC, 0.946
Biologic characterization									
Korfiatis 2016^93^		Glioblastoma	*MGMT* methylation status prediction	155	MRI conventional	SVM, random forest	Texture	Cross‐validation, single center	AUC, 0.85; Sn, 0.803; Sp, 0.813
Zhou 2017^94^		Glioma (WHO grade 3‐4)	*IDH1/IDH2* mutant vs wild type	120	MRI conventional, apparent diffusion maps	Random forest	Histogram, texture, shape	Independent validation with single‐center data	ACC, 89%; AUC, 0.923
Zhang 2017^95^		Glioma (WHO grade 2‐3)	1p/19q Chromosomal status, IDH1/IDH2 mutation status	165	MRI conventional	Logistic regression	VASARI features	Boot‐strap validation, single center	AUC, 0.86
Chang 2018^96^		Glioma (WHO grade 2‐4)	*IDH1/IDH2* mutant vs wild type	496	MRI conventional, apparent diffusion maps	Deep learning ResNet	Histogram, texture, shape	Independent validation with multicenter data	ACC, 89%; AUC, 0.95
Monitoring treatment response									
Larroza 2015^97^		Brain metastases	Classify tumor vs radiation necrosis	73	MRI conventional	SVM	Texture	Cross‐validation, single center	AUC, >0.9
Tiwari 2016^98^		Glioma and brain metastases	Classify tumor vs radiation necrosis	58	MRI conventional	SVM	Intensity, texture	Independent validation with multicenter data	ACC, 80%
Kim 2017^99^		High‐grade glioma	Classify tumor vs radiation necrosis	51	MR diffusion, perfusion, susceptibility weighted maps	Regression	Intensity, histogram	Single‐center, prospective trial without validation	Sn, 71.9%; Sn, 100%; Sp, 100%; ACC, 82.3%
Kebir 2017^100^		High‐grade glioma	Classify tumor vs radiation necrosis	14	FET PET	Unsupervised consensus clustering	Texture	Single‐center, retrospective trial without validation	Sn, 90%; Sp, 75% for detecting true progression; NPV, 75%
Predicting treatment response and survival									
Chang 2016^101^		Glioblastoma	Predict OS	126	MRI conventional, diffusion	Random forest	Shape, intensity histogram, volume, texture	Single‐center data split into training/testing	HR, 3.64 (*P* < .005)
Grossmann 2017^102^		Glioblastoma	Predict PFS and OS	126	MRI conventional	Unsupervised principle component feature selection, random forest supervised training	Shape, volume, texture	Multicenter, phase 2 clinical trial data split into training/testing	OS: HR, 2.5 (*P* = .001); PFS: HR, 4.5; *P* = 2.1 × 10^−5^
Liu 2017^103^		Glioblastoma	Predict OS	117	MRI perfusion	Unsupervised consensus clustering	Histogram of intensity	Single‐center data split into training/testing	HR, >3.0; *P* < .01

Abbreviations: ACC, accuracy; AUC, area under the curve; CNS, central nervous system; FET PET, 18F‐fluoro‐ethyl‐tyrosine positron emission tomography; HGG, high‐grade glioma; HR, hazard ratio; *IDH1/IDH2*, isocitrate dehydrogenase 1/isocitrate dehydrogenase 2; LGG, low‐grade glioma; *MGMT*, O‐6‐methylguanine‐DNA methyltransferase; MRI, magnetic resonance imaging; NPV, negative predictive value; OS, overall survival; PFS, progression‐free survival; ResNet, residual network; Sn, sensitivity; Sp, specificity; SVM, support vector machine; VASARI, Visually Accessible Rembrandt Images; WHO, World Health Organization. ^a^Validation categories included cross‐validation (within own data set), independent validation with single‐center data, and independent validation with multicenter data.

In the setting of gliomas, the most common malignant primary brain tumors in adults, cross‐sectional imaging techniques such as CT and MRI provide high‐resolution spatial information as well as tissue contrast, allowing radiologists to characterize different glioma subtypes and grades. AI can improve the utility of current standard diagnostic imaging techniques by refining the preoperative classification of gliomas beyond what human experts can provide. For example, AI has been applied in the research setting to preoperative MRI to distinguish between low‐grade and high‐grade tumors as well as individual WHO grades by training machine‐learning classifiers using image texture features obtained from spatially coregistered, multimodal MRIs (Table 2).^88^, ^89^, ^90^, ^91^, ^92^, ^93^, ^94^, ^95^, ^96^, ^97^, ^98^, ^99^, ^100^, ^101^, ^102^, ^103^ Furthermore, clinically relevant molecular subtypes of gliomas, such as the presence of an isocitrate dehydrogenase (IDH) mutation, can be identified using machine‐learning methods, including deep CNNs trained on conventional MR images.^94^, ^95^, ^96^

Subtype classification problems are not unique to adult gliomas, however. Conceptually similar work has been done on other brain tumors, in which it has been demonstrated that classification algorithms trained on radiomics features extracted from conventional MRI can generate predictive models for pituitary adenoma subtypes^91^ and pediatric brain tumors (Table 2).^88^, ^89^, ^90^, ^91^, ^92^, ^93^, ^94^, ^95^, ^96^, ^97^, ^98^, ^99^, ^100^, ^101^, ^102^, ^103^

Diagnostic ambiguity also can arise when distinguishing between different tumor types. One key clinical dilemma is when differentiating between primary CNS lymphoma and glioblastoma, which can have similar imaging phenotypes. Radiomics models, using image‐based texture features, have been shown to enhance the differences between glioblastoma and primary CNS lymphoma.^92^, ^104^ Interestingly, a similar diagnostic dilemma often arises when evaluating histopathology slides of these same 2 disease processes; as CNNs are being applied increasingly to histopathology image classifications in research studies across the globe,^105^ we expect robust predictive models to emerge that address this problem as well.

To date, most research applications of AI in brain tumors have focused on addressing challenges in distinguishing between histopathologic and molecular subtypes of brain tumors.^89^, ^92^, ^96^ To accomplish this, AI algorithms are trained using preselected patient populations with the specific tumor subtypes. This approach makes it challenging to integrate diagnostic models into the clinical workflow, because the model’s diagnostic accuracy can be consistent only when the testing population resembles that of the training data. With sufficient training data based on more general patient populations, it is likely that the diagnostic capability of AI will expand to include accuracy differentiation among multiple tumor types as well as nontumor mimickers.

### Tumor Detection and Delineation

Synergistic with accurate diagnostic differentiation between tumor subtypes is the ability of computational algorithms to automatically detect the presence and extent of the tumor itself. On MRI, which is the most common modality of delineating CNS neoplasms, tumors may manifest with variable levels of contrast enhancement or none at all; may be associated with peritumoral edema or hemorrhage; and may blur in margins from adjacent bone, blood vessels, fat, or surgical packing materials. Furthermore, neural response to treatment, also known as pseudoprogression, contributes an additional layer of complexity in discerning tumor from nontumor, as detailed below. Although these features challenge the automatic detection of CNS tumors, the need to develop robust volumetric algorithms for the analysis of tumor and its adjacent microenvironment remains vital.

An escalating cascade of studies and methodologies for the semiautomatic and automatic detection of CNS tumors has been published in recent years, largely applied to conventional MR imaging, but also to PET and ultrasound images.^106^, ^107^, ^108^, ^109^, ^110^, ^111^, ^112^, ^113^ Although they are used most frequently in the exploratory and research setting, semiautomatic algorithms have been applied to treatment planning for stereotactic radiosurgery,^111^ quantitating the volume of residual tumor after surgery,^113^ and tracking tumor growth over time.^109^ One can envision the benefits of a robust, automatic tumor‐detection algorithm in the assessment of patients who have numerous intracranial lesions, such as within the setting of CNS metastases, and their differential growth rate or response to treatment over time. Likewise, in skull‐base lesions, which often are irregularly shaped and extend across intracranial and extracranial compartments, automatic volumetric reconstruction may detect sensitive changes in growth that are missed by the casual observer.

The near universal accessibility of computational tools for image analysis and the sharing of open‐source code by several researchers promises to accelerate the pace of advancement in this field.^114^ In addition, publicly available imaging databases offer powerful resources for hypothesis testing and validation, including the Multimodal Brain Tumor Image Segmentation challenge (BRaTS) data from the Medical Image Computing & Computer Assisted Intervention (MICCAI) group, The Cancer Imaging Archive, and the Ivy Glioblastoma Atlas Project.^115^ Ultimately, the fruit of such efforts hopefully will develop tools that minimize interobserver variability in tracking tumors across time and treatments and extract deeper layers of data beyond radiographic phenotype from routine imaging for CNS tumors.

### Monitoring Response to Treatment

In 20% to 30% of patients with glioblastoma who receive standard, upfront radiation with adjuvant temozolomide, enlargement of contrast‐enhancing lesion(s) that stabilize or resolve without changes in treatment are observed and termed pseudoprogression.^116^ Similarly, approximately 25% of CNS metastases develop necrosis within the irradiated field, manifesting as enlarging enhancement that mimics recurrent tumor after stereotactic radiosurgery of brain metastasis.^116^, ^117^ Although many conventional or advanced imaging techniques have been investigated to distinguish true tumor from treatment‐related changes, it remains challenging to spatially characterize heterogeneous tissues that often contain both viable tumor and treatment‐related changes. Combining multiple imaging features using machine‐learning approaches can improve the ability to construct an accurate tissue classifier that can account for the heterogeneity of treated tumors. Texture features extracted from conventional MRI have been identified to distinguish radionecrosis from recurrent brain tumors.^97^, ^98^ Perfusion‐weighted and susceptibility weighted MRI sequences also can be combined to differentiate recurrence from radionecrosis in patients with high‐grade glioma.^99^ Texture analysis also has been applied to amino acid PET imaging to diagnose pseudoprogression.^100^ To provide a more direct historical correlation of tumor and necrotic tissues, voxel‐based evaluation of MRI coregistered to sites of stereotactic biopsy has resulted in a parametric model that correlates with cell counts of the biopsied specimens.^118^ Overall, most of this research is in the phase of moving from pilot data to validation in clinical trials. Only upon more rigorous proof of the clinical utility of such technology can regulatory approval and commercialization be achieved followed by dissemination into widespread clinical use.

### Biologic Characterization of CNS Tumors: Prospects and Promise

A molecular taxonomy is being defined for the most common CNS tumors with the wide availability and decreasing cost of next‐generation sequencing. Furthermore, it has been observed that molecular signatures confer prognostic implications beyond standard histopathologic classifications, including for adult and pediatric gliomas, meningiomas, pituitary tumors, craniopharyngiomas, medulloblastomas, and ependymomas. These molecular imprints increasingly guide the frequency of surveillance imaging for a tumor, patient consultation for clinical outcome and recurrence risk, and decisions on the type of treatment to administer (eg, radiation or observation).^119^, ^120^, ^121^ However, such information is largely determined only from tissue sampling of the tumor after an intervention. In addition, as with systemic cancers, brain tumors harbor incredible molecular heterogeneity within an individual tumor and on recurrence using multifocal sampling and single‐cell sequencing strategies. Such heterogeneity likely contributes to the limited effectiveness of current pharmacotherapeutics against brain tumors and the perceived acquired resistance after a period of apparent disease control. Therefore, a noninvasive method of tracking tumor genotype over time that can capture the entire landscape of tumor heterogeneity offers appeal.

**Figure 5:**
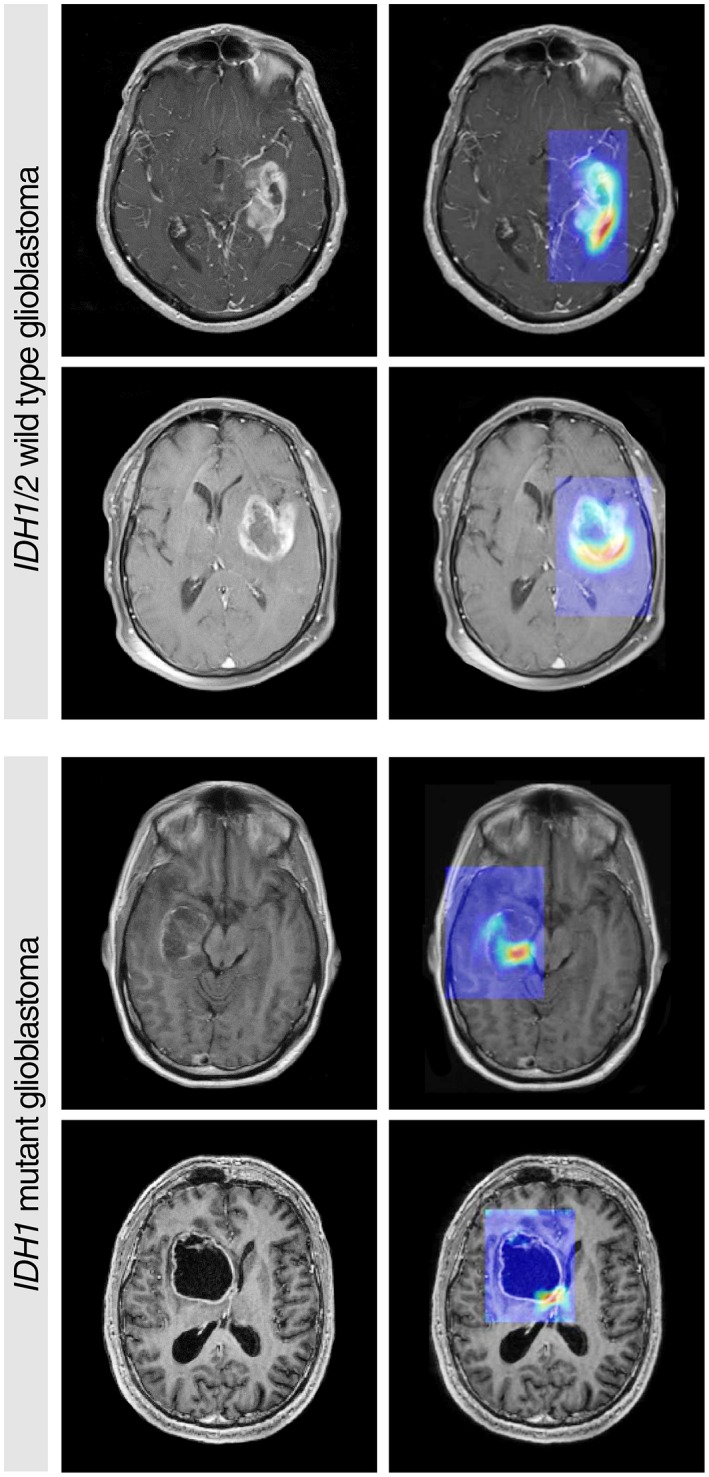
Grad‐CAM Visualizations (Selvaraju et al 2017)^127^ for a Convolutional Neural Network (Chang et al 2018^96^) Applied to 2 Examples of Isocitrate Dehydrogenase 1 (*IDH1*)/*IDH2* Wild‐Type Glioblastoma and 2 Examples of *IDH1*‐Mutant Glioblastoma. Color maps are overlaid on original gadolinium‐enhanced, T1‐weighted magnetic resonance images, with red color weighted to the discriminative regions for IDH status classification.

Radiomic analysis of CNS tumor imaging has the potential to characterize the phenotype of the entire tumor, rather than a core of the tumor, as is frequently sampled for molecular analysis, and provides a noninvasive window into the internal growth pattern of the tumor. Previous works have reported significant connections between imaging features, molecular pathways, and clinical outcomes across brain tumors. The behavior of gliomas is significantly associated with their molecular alterations, especially alterations in *IDH1*/*IDH2*, *EGFR*, O^6^‐methylguanine‐DNA methyltransferase (*MGMT*), and chromosomes 1p and 19q. The WHO recognized the significance of molecular stratification in gliomas in its 2016 update on the classification of gliomas.^122^ Machine‐learning algorithms trained on preoperative MR images have been able to distinguish each of these features with 80% to 95% sensitivity and specificity, including the prediction of glioblastoma subtypes and survival,^123^ *IDH* mutation status in high‐grade and low‐grade gliomas,^95^, ^96^ the presence of chromosome 1p and 19q loss in low‐grade gliomas,^95^, ^124^ MGMT methylation status,^93^ EGFR amplification status,^125^ and the presence of EGFR receptor variant III^126^ as well as *EGFR* extracellular domain mutations (Fig. 5).^96^, ^127^ Moreover, unsupervised deep learning methods are showing promise in discerning molecular subgroups in glioblastoma with differential prognoses.^128^

In meningioma, benign variants (grade I) most commonly carry a mutation in one of several putative oncogenic drivers, whereas high‐grade variants (grade II‐III tumors) harbor a variable number of chromosomal alterations. Radiomic analysis of preoperative MRIs from patients with meningioma revealed the ability of computer‐extracted imaging features to strongly associate with meningioma grade^89^ and also with certain genomic features (W.L.B. and H.J.W.L.A. et al, unpublished data). In addition, quantitative radiomic features could discern subtleties, such as the number of atypical features associated with grade I meningiomas, beyond the capacity of qualitative radiologist‐rated imaging features.^89^

Similar radiomic analyses are developing for pituitary tumors,^91^ craniopharyngiomas, chordomas, and other CNS tumors. Beyond single‐tumor subtype analysis, future efforts need to improve the accuracy and sensitivity such that such methods can be applied to the clinical setting with confidence, derive more nuanced molecular signatures beyond that of a single or dual marker, and accommodate the artifacts associated with recurrent and post‐treatment disease states to allow for truly longitudinal application of radiomics throughout the course of patients who have CNS tumors.

### Clinical Trial Applications

Predictive biomarkers can have important roles in clinical trials because of their ability to select patients who are more likely to respond to treatment and thereby improve the chance of detecting clinical benefit and lowering the risk of drug toxicity from ineffective therapies. The best known predictive biomarkers for the treatment of glioblastoma is MGMT promoter status, in which methylated tumor subtypes have shown greater response to alkylating agents.^129^, ^130^ Recently, the antiangiogenic treatment of newly diagnosed glioblastoma was evaluated in 2 phase 3 clinical trials in which bevacizumab, an antibody targeting VEGF, did not result in improved OS when added to the standard treatment.^131^, ^132^ Currently, there is no clinically useful molecular marker predictive of treatment response for antiangiogenic therapy. Imaging‐based biomarkers of treatment response prediction for newly diagnosed, recurrent glioblastoma have been investigated using both conventional and advanced modalities. Radiomic imaging predictors of response based on conventional imaging features have been identified using a retrospective, single‐center data set of patients with recurrent glioblastoma who received bevacizumab treatment. In a retrospective evaluation of single‐institution and multi‐institutional, single‐arm data sets, radiomic models were constructed using conventional and diffusion MRI features to differentiate long‐term from short‐term survivors.^101^, ^102^ Unsupervised clusters of radiomic features based on nonparametric parameters of preoperative perfusion MRI were first extracted independently from 2 data sets of patients with glioblastoma, and the feature clusters subsequently were combined and evaluated for their association with patient survival outcome.^103^ The radiomic cluster that was associated with poor survival (HR, >3.0) was associated with mutations in the angiogenesis and hypoxia pathways. These preliminary investigations were based on patients receiving therapy without the availability of a control treatment arm and thus only establish the prognostic values of these imaging markers.

There are several advantages of using clinical trial data, both retrospectively and prospectively, to screen and validate radiomic biomarkers. Because these patient populations are relatively uniform (including treatment regimen type, dose, and duration as well as imaging assessment timing and frequency during the pretreatment and on‐treatment periods), the predictive accuracy for patient outcome will likely improve. The predictive models constructed in this setting can be applied more readily to future prospective trials that use similar protocols. Recent efforts to standardize imaging acquisition protocols for brain tumor trials also should increase the generalizability of radiomic models to different clinical trials and to actual clinical implementation.^133^

## Breast Cancer Imaging

Breast cancer is the most commonly diagnosed cancer and the second most common cause of cancer death in US women.^31^ The 5‐year survival rates for breast cancer have improved tremendously since the 1980s, likely because of the significant uptake of mammographic screening as well as improvements in breast cancer treatment. Breast cancer is a heterogeneous disease, and tumors vary with respect to etiology, prognosis, and response to therapy. The presence of the estrogen receptor (ER) is important for responses to specific treatments (eg, tamoxifen for patients with ER‐positive disease) and prognosis (poorer outcomes for those with ER‐negative disease) and may define etiologic subtypes. Triple‐negative breast cancers are ER‐negative, progesterone receptor (PR)–negative, and human epidermal growth factor receptor 2 (HER2)–negative. They do not present with the typical signs of malignancy on standard mammography,^134^ are more likely to be detected as interval and high‐grade tumors, and have a poor 5‐year survival rate.

Advances in both imaging and computers have synergistically led to a rapid rise in the potential use of AI in various tasks in breast imaging, such as risk assessment, detection, diagnosis, prognosis, and response to therapy (Table 3).^135^, ^136^, ^137^, ^138^, ^139^, ^140^, ^141^, ^142^, ^143^, ^144^, ^145^, ^146^, ^147^, ^148^, ^149^, ^150^

**Table 3:** Summary of Key Studies on Imaging Characterization of Breast Lesions, Including Detection, Diagnosis, Biologic Characterization, and Predicting Prognosis and Treatment Response

REFERENCE	APPLICATION	NO. OF CASES	IMAGING MODALITY	MACHINE LEARNING ALGORITHM (IF APPLICABLE)	IMAGING/RADIOMIC FEATURE TYPE	RESULTS	
Detection							
Zhang 1994^135^	Microcalcification detection	34	Mammography	Convolutional neural networks	Deep learning characterization followed by conventional image analysis	AUC, 0.91	
Karssemeijer 2006^136^	Mass lesions	500	Mammography	Engineered algorithms	Engineered algorithms	Performance similar to radiology	
Reiner 2006^137^	Mass lesions	21	Breast tomosynthesis	Engineered algorithms	Engineered algorithms	Sn, 90%	
Sahiner 2012^138^	Microcalcifications	72	Breast tomosynthesis	Engineered algorithms	Engineered algorithms	Sn, 90%	
Diagnosis							
Gilhuijs 1998^139^	Mass lesions	27	DCE‐MRI	Engineered algorithms	Size, shape, kinetics	AUC, 0.96	
Jiang 1999^140^	Microcalcifications	104	Mammography	Engineered algorithms	Size and shape of individual microcalcifications and clusters	AUC, 0.75	
Chen 2007^141^	Mass lesions	121	DCE‐MRI	Engineered algorithms	Uptake heterogeneity in cancer tumors via 3D texture analysis	3D better compared with 2D analysis	
Booshan 2010^142^	Differentiate benign vs DCIS vs IDC	353	DCE‐MRI	Bayesian neural networks	Size, shape, margin morphology, texture (uptake heterogeneity), kinetics, variance kinetics	AUC, 0.79‐0.85	
Jamieson 2010^143^	Mass lesions	1126	Multimodality: Mammography, breast ultrasound, and breast DCE‐MRI	t‐SNE followed by Bayesian neural networks	Multiradiomic features in nonsupervised data mining	AUC, 0.88	
Nielsen 2011^144^	Breast cancer risk	495	Mammography	—	Texture analysis	AUC, 0.57‐0.66	
Huynh 2016^145^	Mass lesions	219	Mammography	Deep learning	Feature extracted from transfer learning from pretrained CNN	AUC, 0.81	
Andropova 2017^146^	Mass lesions	1125	Multimodality: Mammography, breast ultrasound, and breast DCE‐MRI	Deep learning	Fusion of human‐engineered computer features and those feature extracted from transfer learning from pretrained CNN	AUC: DCE‐MRI, 0.89; FFDM, 0.86; ultrasound, 0.90	
Biologic characterization							
Gierach 2014^147^	*BRCA1/2* mutation status	237	Mammography	Bayesian artificial neural network	Texture analysis	AUC, 0.68‐0.72	
Li 2016^148^	Molecular subtype classification	91 (from TCGA)	DCE‐MRI	Engineered features, linear discriminant analysis	Multiradiomic tumor signature, including size, shape, margin morphology, texture (uptake heterogeneity), kinetics, variance kinetics	AUC, 0.65‐0.89	
Li 2017^149^	*BRCA1/2* mutation status	456	Mammography	CNNs, computerized radiographic texture analysis, SVM	Texture analysis and deep learning	AUC, 0.73‐0.86	
Predicting treatment response and prognosis							
Drukker 2018^150^	Prediction of recurrence‐free survival	284 (from ACRIN 6657)	DCE‐MRI	—	.Most‐enhancing tumor volume	HR, 2.28‐4.81	

Abbreviations: 2D, 2‐dimensional; 3D, 3‐dimensional; ACC, accuracy; ACRIN, American College of Radiology Imaging Network; AUC, area under the curve; CNN, convolutional neural networks; DCE‐MRI, dynamic contrast‐enhanced magnetic resonance imaging; DCIS, ductal carcinoma in situ; FFDM, full‐field digital mammography; HR, hazard ratio; IDC, invasive ductal carcinoma; Sn, sensitivity; Sp, specificity; SVM, support vector machine; TCGA, The Cancer Genome Atlas; t‐SNE, t‐distributed stochastic neighbor embedding.

### Breast Cancer Screening: Breast Imaging Reporting and Data System Analog to Digital

CADe and CADx in breast cancer imaging have been under development for decades.^151^, ^152^, ^153^ CADe systems specifically for screening mammography interpretation have been in routine clinical use since the late 1990s.^153^, ^154^ The detection of cancer by radiologists is limited by the presence of structure noise (camouflaging normal anatomic background), incomplete visual search patterns, fatigue, distractions, the assessment of subtle and/or complex disease states, vast amounts of image data, and the physical quality of the breast image itself. In computer‐aided detection, the computer aims to locate suspect lesions, leaving the classification to the radiologist.

Although CADe continues to be developed for screening mammography, investigators also have sought to automate the detection of breast lesions on 3D ultrasound, breast MRI, and breast tomosynthesis images by incorporating predefined algorithms as well as novel deep learning methods.^155^, ^156^, ^157^, ^158^ The motivation for computerized detection on 3D breast images arose with the arrival of 3D ultrasound and MRI for use as adjunct imaging for screening women with dense breast tissue.^150^

CNNs have been used in medical image analysis since the early 1990s for the detection of microcalcifications in digitized mammograms^135^ as well as for distinguishing between biopsy‐proven masses and normal tissue on mammograms.^159^ More recently, deep learning methods have allowed for the computer‐aided detection of breast lesions in breast MRI, ultrasound, and mammography.^155^, ^156^, ^157^, ^158^

### Breast Cancer Risk Assessment: Density and Parenchyma

Computer vision techniques have been developed to extract the density and characteristics of the parenchyma patterns on breast images to yield quantitative biomarkers for use in breast cancer risk prediction and, ultimately, in personalized screening regimes.

Both area‐based and volumetric‐based assessments of density are used to estimate mammographic density, because increased density serves as a breast cancer risk factor as well as provides a masking effect that obscures lesions.^160^, ^161^, ^162^ Breast density refers to the amount of fibroglandular tissue in the breast relative to the amount of fatty tissue. In full‐field digital mammography (FFDM), these tissue types are distinguishable, because fibroglandular tissue attenuates x‐rays much more than fat tissue. Because FFDMs are 2D projections of the breast, 3D percentage density values are estimated.

In addition to breast density, there is also evidence that the variability in parenchymal patterns (eg, characterizing the spatial distribution of dense tissue) also is related to breast cancer risk. By using radiomic texture analysis, investigators have characterized the spatial distribution of the gray‐scale levels within regions on FFDM when a skewness measure was incorporated into the analysis of mammograms to describe the density variation.^160^ Others have used texture analysis and deep learning to discriminate BRCA1/BRCA2 gene mutation carriers (or women with breast cancer in the contralateral breast) from women at low risk of breast cancer and, using almost 500 cases, found that women at high risk of breast cancer have dense breasts with parenchymal patterns that are coarse and low in contrast (AUC, approximately 0.82).^149^, ^163^, ^164^ Further efforts have applied texture analysis to breast tomosynthesis images to characterize the parenchyma pattern for ultimate use in breast cancer risk estimation, with preliminary results indicating that texture features correlated better with breast density on breast tomosynthesis (*P* = .003 in regression analysis) than on digital mammograms.^165^

In addition, the characterization of breast parenchymal patterns has also been extended to breast parenchymal enhancement (BPE) on dynamic contrast‐enhanced (DCE)‐MRI.^163^, ^164^, ^166^ In a limited data set of 50 BRCA1/BRCA2 carriers, quantitative measures of BPE were associated with the presence of breast cancer, and relative changes in BPE percentages were predictive of breast cancer development after risk‐reducing salpingo‐oophorectomy (*P* < .05).^167^ Deep learning methods are increasingly being evaluated to assess breast density as well as parenchymal characterization, an example of which includes the performance assessment of transfer learning in the distinction between women at normal risk of breast cancer and those at high risk based on their BRCA1/BRCA2 status.^149^

### AI to Improve Breast Cancer Diagnosis

Since the 1980s, various investigators have been developing machine learning techniques for CADx in the task of distinguishing between malignant and benign breast lesions.^168^ These AI methods for CADx involve the automatic characterization of a tumor, which is indicated initially by either a radiologist or a computer. The computer characterizes the suspicious region or lesion and/or estimates its probability of disease, leaving patient management to the physician.

With the application of AI methods to breast image data, characteristics of tumor size, shape, morphology, texture, and kinetics can be quantitatively obtained. For example, use of the dynamic assessment of contrast uptake on breast MRI allows investigators to quantify cancers in terms of heterogeneity, yielding phenotypes of spatial features and dynamic characteristics.^141^, ^169^ For example, entropy is a mathematical descriptor of randomness and provides information on how heterogeneous the pattern is within the tumor, thus describing the heterogeneous pattern of the vascular system uptake (ie, contrast uptake) within tumors imaged on contrast‐enhanced breast MRI. Such analyses potentially could reflect the heterogeneous nature of angiogenesis and treatment susceptibility, as shown by the NCI’s The Cancer Genome Atlas (TCGA) Breast Cancer Phenotype Group.^170^

With CADx, both predefined and deep‐learned algorithms have been evaluated. It is interesting to note that investigators have shown that the use of either human‐engineered or deep learning features perform well in the classification of breast lesions in the task of distinguishing between malignant and benign lesions and that the “fusion” of the 2 methods can yield a statistically significant improvement in performance.^145^, ^146^ Across all 3 breast‐imaging modalities (690 DCE‐MRI cases, 245 FFDM cases, and 1125 ultrasound cases), the “fusion” classifier performed best, indicating the potential for the complimentary use of both engineered and deep learning tumor features in diagnostic breast cancer workup (DCE‐MRI: AUC = 0.89 [standard error = 0.01]; FFDM: AUC = 0.86 [standard error = 0.01]; and ultrasound: AUC = 0.90 [standard error = 0.01]).^146^ Other investigators have used transfer learning with CNNs pretrained on 2282 digitized screen‐films and FFDMs for use in characterizing tumors on 324 breast tomosynthesis volumes, which demonstrated the ability to transfer knowledge of the imaged patterns between the imaging modalities.^171^

### Predictive Image‐Based Biomarkers

Beyond CADe and CADx,^2^ other AI applications in breast imaging include assessing molecular subtypes, prognosis, and therapeutic response by yielding predictive image‐based phenotypes of breast cancer for precision medicine. A major area of interest within breast cancer research is the attempt to understand relationships between the macroscopic appearance of the tumor and its environment. These relationships can be extracted from clinical breast images and the biologic indicators of risk, prognosis, or treatment response. Such an effective development of biomarkers benefits from the integration of information from multiple patient examinations (ie, clinical, molecular, imaging, and genomic data; ie, the other “‐omics” that often are obtained during diagnostic workup and subsequent biopsies).

**Figure 6:**
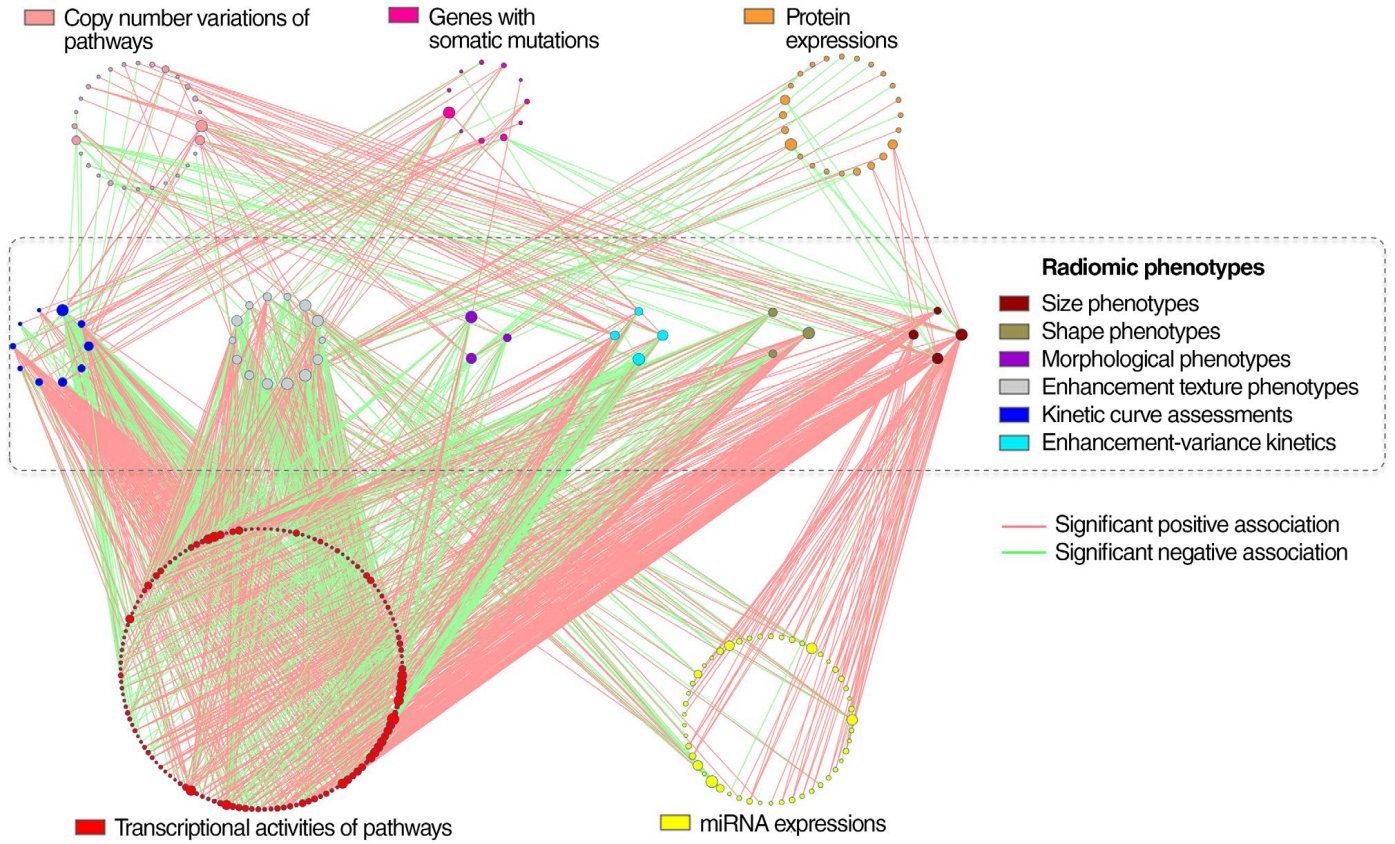
Significant Associations Between Genomic Features and Radiomic Phenotypes in Breast Carcinoma Imaged With Magnetic Resonance Imaging. Gene‐set enrichment analysis (GSEA) and linear regression analysis were combined to associate genomic features, including microRNA (miRNA) expression, protein expression, and gene somatic mutations among others, with 6 categories of radiomic phenotypes. In this figure, each node represents a genomic feature or a radiomic phenotype. Each line is an identified statistically significant association, whereas nonsignificant associations are not depicted. Node size is proportional to its connectivity relative to other nodes in the category. Reprinted with permission from Maryellen L. Giger, University of Chicago (Zhu Y, Li H, Guo W, et al. Deciphering genomic underpinnings of quantitative MRI‐based radiomic phenotypes of invasive breast carcinoma [serial online]. *Sci Rep*. 2015;5:17787.^170^).

In one collaborative effort through the NCI’s TCGA Breast Phenotype Group, multidisciplinary investigators phenotypically characterized 84 solid breast tumors to gain image‐based information on the underlying molecular characteristics and gene expression profiles (Fig. 6).^170^, ^172^ Statistically significant associations were observed between enhancement texture (entropy) and molecular subtypes (normal‐like, luminal A, luminal B, HER2‐enriched, basal‐like), even after controlling for tumor size (*P* = .04 for lesions ≤2 cm; *P* = .02 for lesions from 42 to ≤5 cm). MRI‐genomic associations also were unveiled, furthering the understanding of genetic mechanisms that regulate the development of tumor phenotypes.^146^, ^170^

With regard to predicting a patient’s response to a particular therapeutic treatment, for example, the semimanual delineation of functional tumor volume from breast MRI (141 women: 40 with a recurrence and 101 without) was identified as a predictor of recurrence‐free survival in patients receiving neoadjuvant therapy in the American College of Radiology Imaging Network (ACRIN) study 6657,^173^ with demonstrated potential for automation.^174^

## Prostate Cancer Imaging

Prostate cancer is the most frequently diagnosed, noncutaneous male malignancy and the second leading cause of cancer‐related mortality among men in the United States.^31^ Statistics of prostate cancer frequency, morbidity, and mortality can be examined in many different ways. It is a very common cancer, as it is a “tumor of aging,” but it has a very low disease‐specific mortality, all of which reinforce its characterization as a complex public health concern that impacts a large population. Although prostate cancer is a serious disease, most men diagnosed with prostate cancer do not die of it.^175^ The 5‐year survival rate for patients with prostate cancer ranges from approximately 30% in patients with metastatic disease to 100% in patients with localized disease. The key clinical problems in prostate cancer diagnosis today include: 1) overdiagnosis and overtreatment resulting from an inability to predict the aggressiveness and risk of a given cancer; and 2) inadequate targeted biopsy sampling, leading to misdiagnosis and to disease progression in men with seemingly low‐risk prostate cancer. In a meta‐analysis,^176^ the reported rate of overdiagnosis of nonclinically significant prostate cancer was as high as 67%, leading to unnecessary treatment and associated morbidity. Because of this range of clinical behavior, it is necessary to differentiate men who have clinically significant tumors (those with a biopsy Gleason score 7 and/or pathologic volume 0.5 mL)^177^ as candidates for therapy from those who have clinically insignificant tumors and can safely undergo active surveillance. It has been noted that potential survival benefits from aggressively treating early‐stage prostate cancer are undermined by harm from the unnecessary treatment of indolent disease.

The biological heterogeneity of prostate cancer leads to different clinical outcomes, ranging from indolent to highly aggressive tumors with high morbidity and mortality, and differences in therapy planning, therapy response, and prognosis of patients. This is reflected by the incorporation of genomic profiling in the National Comprehensive Cancer Network guidelines, including Decipher (GenomeDx Biosciences, San Diego, California), Oncotype DX Prostate (Genomic Health Inc, Redwood City, California), Prolaris (Myriad Genetics Inc, Salt Lake City, Utah), and others. In parallel with molecular characterization, AI also has the potential to empower clinicians in the detection, localization, characterization, staging, and monitoring of prostate cancer. There are no widespread multicenter trials as yet, and therefore much of the initial work is limited to single‐center, single‐algorithm analyses and on small data sets. However, some groups, such as the National Institutes of Health and MICCAI, are developing infrastructure to allow larger, well annotated data sets to become available for AI development.

Computational methods mostly based on supervised machine learning have been successfully applied to imaging modalities such as MRI and ultrasound to detect suspicious lesions and differentiate clinically significant cancers from the rest. The recent application of deep learning in prostate cancer screening and aggressive cancer diagnosis has produced promising results.

Multiparametric magnetic resonance imaging (mpMRI) provides the required soft‐tissue contrast for the detection and localization of suspicious clinically significant prostate lesions and gives information regarding tissue anatomy, function, and characteristics. Importantly, it has superior capabilities to detect the so‐called “clinically significant” disease—one with a Gleason pattern of 4 or higher (Gleason score ≥7) and/or a tumor volume >0.5 cm^3^. Recent years have seen a growth in the volume of mpMRI examination of prostate cancer because of its ability to detect these lesions and allow targeted biopsy sampling. A large population study from the United Kingdom suggested that the use of mpMRI as a triage before primary biopsy can reduce the number of unnecessary biopsies by one‐quarter and can decrease overdiagnosis of clinically insignificant disease.^178^ This was further validated in data sets that were smaller than would be considered optimal. In the multinational PRECISION study of 500 patients,^179^ men randomized to undergo mpMRI before biopsy experienced a significant increase in the detection of clinically significant disease over the current standard of care, which uses a 10‐core to 12‐core transrectal ultrasound‐guided biopsy (38% vs 26%).

The growing trend toward mpMRI has introduced a demand for experienced radiologists to interpret the exploding volumes of oncological prostate MRIs. Furthermore, reading challenging cases and reducing the high rate of interobserver disagreements on findings is a remaining challenge for prostate MRI. In 2015, the European Society of Urogenital Radiology, the American College of Radiology, and the AdmeTech Foundation published the second version of the Prostate Imaging Reporting and Data System (PI‐RADS). This provides guidelines for radiologists in reading and interpreting the prostate mpMRI, which aim to increase the consistency of the interpretation and communication of mpMRI findings. Over the past 10 years, AI models have been developed as CADe and CADx systems to detect, localize, and characterize prostate tumors.^180^ In conjunction with PI‐RADS, accurate CAD systems can increase the interrater reliability and improve the diagnostic accuracy of mpMRI reading and interpretation.^181^ In preliminary analyses, it has been shown that the addition of a CADx system can improve the performance of radiologists in prostate cancer interpretation.^182^, ^183^

Preliminary work in mpMRI CADx systems focused primarily on classic, supervised machine learning methodologies, including combinations of feature extractors and shallow classifiers. In this category of AI systems, feature engineering plays a central role in the overall performance of the CAD system. Combinations of CADe and CADx systems have been reported that use intensity, anatomic, pharmacokinetic (pharmacokinetic modeling), texture, and blobness features.^184^ Pharmacokinetics are the detailed metrics that can be extracted from a time‐signal analysis of intravenous contrast passing through a given volume of tissue. They include parameters such as wash‐in and wash‐out. Texture features also are signal‐based and depend heavily on the imaging technique. Others have use intensity features calculated from mpMRI sequences, including T2‐weighted MRI, the apparent diffusion coefficient, high b‐values, diffusion‐weighted MRI, and T2 estimation by proton density mapping,^184^ or they have only used features extracted from pharmacokinetic analysis and diffusion tensor imaging parameter maps.185 Similar image‐based features were included in CAD systems,^186^, ^187^, ^188^, ^189^ and many of these systems use support vector machines for classification.^185^, ^187^, ^190^, ^191^, ^192^

In the past years, deep learning networks, and particularly CNNs, have been revolutionizing investigative research into prostate cancer detection and diagnosis. These methods use different modality types, CNN architectures, and learning procedures to train deep networks for prostate cancer classification and have achieved state‐of‐the‐art performance. Some investigators use CNNs to classify MRI findings with an auto‐windowing mechanism to overcome the high dynamic range of MR images and normalization,^193^ whereas others use different combinations of mpMRI images by stacking each modality as a 2D channel of RGB images and use them as training examples.^194^, ^195^ Furthermore, 3D CNNs can be designed that use specific MRI‐based parameters such as apparent diffusion coefficient, high b‐value, and volume transfer constant (K^trans^) modalities.^196^

Deep learning systems have been applied to localize and classify prostate lesions at the same time.^197^ Both de novo training^194^, ^196^, ^197^ and transfer learning of pretrained models^195^ have been successful for training CNNs for prostate cancer diagnosis in MRI. The explicit addition of anatomically aware features to the last layers of CNNs has been used successfully to boost their performance.^193^, ^196^ In addition to MRI, AI techniques have achieved promising results by incorporating ultrasound data, specifically radiofrequency, for prostate cancer classification. Here again, both classic machine learning approaches^198^, ^199^ and deep learning^200^ have been used to train classifiers to grade prostate cancer in temporal ultrasound data.

The results of the ongoing research into the use of AI for the detection and characterization of prostate cancer are promising and demonstrate ongoing improvement. The recent body of research in prostate cancer image analysis reveals a transition from feature engineering and classic machine learning methods toward deep learning and the use of large training sets. Unlike lung and breast cancers, clinical routines in prostate cancer have not yet adopted regulated CAD systems. However, the recently achieved results of deep learning techniques on midsize data sets, such as the PROSTATEx benchmark, are promising. It is now evident that there has been a rapid growth in prostate MR examination volumes worldwide and increasing demand for accurate interpretations. Accurate CAD systems will improve the diagnostic accuracy of prostate MRI readings, which will result in better care for individual patients, because fewer patients with benign and indolent tumors (false‐positives) will need to undergo invasive biopsy and/or radical prostatectomy procedures, which can lower their quality of life. Conversely, early detection of prostate cancer improves the prognosis of patients who have clinically significant prostate cancer (Gleason pattern 4). Computer‐assisted detection and diagnosis systems for prostate cancer help clinicians by potentially reducing the chances of either missing or overdiagnosing suspicious targets on diagnostic MRIs, although this merits additional validation in trials before routine clinical incorporation.

## Challenges and Future Directions

Despite the reported successes of AI within cancer imaging, several limitations and hurdles must be overcome before widespread clinical adoption. With the increasing demand for CT^201^ and MR^202^ imaging, care providers are constantly generating large amounts of data. Standards, including the Picture Archiving and Communication System (PACS) and the Digital Imaging and Communications in Medicine (DICOM), have ensured that these data are organized for easy access and retrieval. However, such data are rarely curated in terms of labeling, annotations, segmentations, quality assurance, or fitness for the problem at hand. The curation of medical data represents a major obstacle in developing automated clinical solutions, because it requires trained professionals, making the process expensive in both time and cost. These issues are exacerbated by data‐hungry methods, including deep neural networks. Unsupervised^203^ and self‐supervised^204^ methods do not require explicit labeling and hence promise to alleviate some of these issues, whereas synthetic data^205^ can potentially enable a faster route toward curation, address the inevitable class imbalance, and mitigate patient privacy concerns. Standardized benchmarking is of particular importance in the medical domain, especially given the multitude of imaging modalities and anatomic sites, as well as acquisition standards and hardware. The research community has yet to reach a consensus on specific data sets that can be used for comparing and contrasting efforts in terms of performance, generalizability, and reproducibility, although the volume of medical data being made public is an encouraging move forward.^206^ Furthermore, access to available data sets should be improved to promote intellectual collaboration. Institutional, professional, and government groups should be encouraged to share validated data to support the development of AI algorithms, which requires overcoming certain fundamental technical, legal, and perhaps ethical concerns.^207^ For example, the National Institutes of Health recently shared chest x‐ray and CT repositories to help AI scientists.^208^ Such efforts bear expansion to a much wider audience across disease states.

Another limitation includes the interpretability of AI and the ability to interrogate such methods for reasons behind a specific outcome, as well as the anticipation of failures. Although the current state of research has prioritized performance gains over explainability and transparency, the interpretability of AI is an active area of research.^209^ The benefits of trust and transparency in AI systems will differ based on their performance, allowing for the identification of failures when AI is subhuman and, consequently, transforming superhuman AI into a learning resource. From a legal standpoint, policy makers have taken note: discussions around improving AI accountability through explanations have recently been debated in the EU General Data Protection Regulation^210^ and continue to surface in sensitive applications for which an explanation is currently required under the law, or is anticipated to be required in the near future.

From an ethical perspective, a question poses itself: What would a Hippocratic Oath for clinically deployed AI systems be, and how would it be enforced? Although data curation and modeling practices are biased in nature, because they take into account specific patient cohorts, a conscious effort must be put into understanding exactly who will be the ultimate beneficiaries and stakeholders of such technology. Algorithms can be unethical by design^211^ and might exacerbate the already existing tension between providing care and turning profits. In addition, a safeguard against “learned helplessness” must be used as a means to curb high reliance on automation and the ultimate abandonment of common sense. Finally, automated systems also might challenge the dynamics of responsibility within the doctor‐patient relationship, as well as the expectation of confidentiality.^212^

In terms of regulatory aspects, the US Food and Drug Administration has been regulating automated clinical decision‐making systems since the 1990s.^213^ With the advent of new prediction techniques, including deep learning, predictive models seeking approval must be further scrutinized in terms of the ground truth data used in training them, their intended use cases, and their generalizability and robustness against edge cases, as well as their life‐long learning aspects, as they are continuously updated with more learning and more data. It is likely that AI application software will need to meet rigorous testing that is mandated for new submissions for regulatory approval, including quality control and risk assessment. Because cloud computing and virtualization are being used increasingly to process medical data, health care information technology is gradually becoming part of the “big data” revolution.^214^ This offers a fertile environment for incorporating state‐of‐the‐art AI systems that often are distributed. Nevertheless, it raises data security and privacy concerns, because maintaining Health Insurance Portability and Accountability Act compliance is essential. Current cyber security research starts to offer solutions, including cryptonets, in which homomorphic encryption allows neural networks to run training and inference on encrypted data.^215^

Today’s diagnostic paradigm in medicine focuses on the detection of visually recognizable findings that suggest discrete pathologies in images. However, the focus of such detection of singular disease processes may miss concurrent conditions in an individual as a whole. Imaging methods, from simple x‐rays to advanced, cross‐sectional imaging methods such as MRI or CT, provide the opportunity to assess cancer in the context of its surrounding organ system.

With the integration of AI, complex assessments of biological networks may have a profound impact on the assessment of response and prognosis and treatment planning. In addition to the finding of a neoplasm, imaging may detect changes in the adjacent or distant organs beyond the tumor that alter patient susceptibility to systemic morbidities, which ultimately can contribute to mortality. This may occur as a byproduct of disease progression itself or as a byproduct of treatment, such as radiation or chemotherapy. For example, in patients undergoing treatment for thoracic or breast cancer, chemotherapy may lead to myocardial damage, whereas radiation therapy promotes advanced coronary atherosclerosis; patients who survive cancer also experience a high rate of cardiovascular events. Collectively, these cardiotoxicities may confer a signal on routine imaging during the monitoring of cancer and be detected in earlier stages of development with comprehensive analytical systems that capture the diorama of disease processes. Initial concepts to apply AI to this clinical scenario stemmed from the finding that thoracic cancers and cardiovascular pathology are adjacent to each other and may be detected simultaneously (ie, coronary calcification or pericardial fat on chest CT). The development of automated, AI‐based detection and quantification algorithms therefore would enable the assessment of cardiometabolic markers without the need for additional imaging. In this manner, the role of AI can be extended to screening by simultaneously evaluating additional risks from the same data source. Because health care ultimately aims to prevent disease, the generation of accurate risk models is essential in guiding actionable risk‐modification strategies.

Although AI can detect incidental findings that may be clinically beneficial, these findings also may be clinically irreverent and, if not carefully framed in the correct clinical context, may increase patient stress, health care costs, and undesired side effects from treatment. It is likely that, during the early phase of AI, when human experts will continue to play key roles in gatekeeping AI’s output, the majority of incidental findings detected by AI will still be evaluated by humans to discern whether or not they are clinically significant in the same manner as when humans detected incidental findings. Over time, as AI systems mature, these incidental findings may become part of standard data evaluation and reporting, the same way primary lesions are evaluated and reported in the patient’s clinical context.

In addition, imaging is not an isolated measure of disease. Increasingly, it is appreciated that the molecular signatures of cancers, including noninvasive blood biomarkers of tumor, socioeconomic status, and even social networks, have an impact on the outcome of patients with cancer. Sources of data also are rapidly expanding beyond direct medical testing and include input from wearables, mobile phones, social media, unstructured electronic health records, and other components of the digital age. AI is well suited to integrate parallel streams of information—biological, demographic, and social—over time to improve predictive models for patient outcome.

As the power and potential of AI are increasingly proven, multiple directions remain for AI to transition into routine clinical practice. For imaging analysis, the accuracy and predictive power of AI methodologies need significant improvement and a demonstration of comparable efficacy, or better, than human experts in controlled studies if they are to be poised to supplant clinician workflows. This shows initial promise in several disease conditions but requires additional proof of clinical utility in prospective trials and education of physicians, technologists, and physicists to incorporate into widespread use.^216^, ^217^ Although there likely will always be a “black box” for human experts in viewing AI‐generated results, data visualization tools are increasingly available to allow some degree of visual understanding of how algorithms make decisions.^127^

The curation of comprehensive data sets and outcomes that incorporate both disease‐related and unrelated elements also will help train and expand AI systems to account for risks beyond cancer itself. In global settings with limited access to expert clinicians or exposure to uncommon pathologies, AI may offer a repertoire of “expert” experiences in disease interpretation. Conversely, strategies that predict outcomes without a ground truth provided by human experts may disrupt the traditional workflow familiar to clinicians and patients today.^218^ Furthermore, the increased incorporation of AI in monitoring health resources and outcomes likely will improve efficiency and reduce cost. As with any new innovative technology, the possibilities for development reside beyond current imagination.
